# Performance Evaluation of Relay Selection Schemes in Beacon-Assisted Dual-Hop Cognitive Radio Wireless Sensor Networks under Impact of Hardware Noises

**DOI:** 10.3390/s18061843

**Published:** 2018-06-05

**Authors:** Tran Dinh Hieu, Tran Trung Duy, Le The Dung, Seong Gon Choi

**Affiliations:** 1 Department of Radio and Communication Engineering, Chungbuk National University, Cheongju City 362763, Korea; trandinhhieu1989@gmail.com (T.D.H.); dung.t.le@ieee.org (L.T.D.); 2 Posts Department of Telecommunications, Posts and Telecommunications Institute of Technology, Ho Chi Minh City 70000, Vietnam; trantrungduy@ptithcm.edu.vn

**Keywords:** energy harvesting, power beacon, decode-and-forward (DF), partial relay selection, opportunistic relay selection, underlay cognitive radio, hardware impairments

## Abstract

To solve the problem of energy constraints and spectrum scarcity for cognitive radio wireless sensor networks (CR-WSNs), an underlay decode-and-forward relaying scheme is considered, where the energy constrained secondary source and relay nodes are capable of harvesting energy from a multi-antenna power beacon (PB) and using that harvested energy to forward the source information to the destination. Based on the time switching receiver architecture, three relaying protocols, namely, hybrid partial relay selection (H-PRS), conventional opportunistic relay selection (C-ORS), and best opportunistic relay selection (B-ORS) protocols are considered to enhance the end-to-end performance under the joint impact of maximal interference constraint and transceiver hardware impairments. For performance evaluation and comparison, we derive the exact and asymptotic closed-form expressions of outage probability (OP) and throughput (TP) to provide significant insights into the impact of our proposed protocols on the system performance over Rayleigh fading channel. Finally, simulation results validate the theoretical results.

## 1. Introduction

In wireless sensor networks (WSNs), energy is one of the most critical resources because sensors are often low-cost, energy-constrained, resource-constrained nodes [1,2]. The energy harvesting (EH) technique [3,4] has been considered as a viable solution to prolong battery lifetime, improve network performance, and provide green communication for WSNs. Therefore, it has received significant interest from the wireless communication community. Besides conventional EH techniques powered by external energy sources such as solar, wind energy, piezoelectric shoe inserts, thermoelectricity, acoustic noise, etc. [5,6,7], radio frequency (RF) energy harvesting (EH) has recently become a promising technique for WSNs since it allows information and energy to be transmitted simultaneously [8,9,10,11,12,13]. In [8], the authors first dealt with the fundamental trade-off between transmitting energy and information at the same time over single input single output (SISO) additive white Gaussian noise (AWGN) channels. Based on these pioneering works, Refs. [9,10] proposed more practical designs, by assuming that the receivers are capable of performing EH and information decoding separately. Zhang and Ho [9] studied multiple input multiple output (MIMO) transmission with practical designs that separate the operation of information decoding and EH receivers. Based on the time switching (TS) and power switching (PS) receiver architectures, Refs. [10,11] proposed two relaying protocols, namely, time switching-based relaying (TSR) and power switching-based relaying (PSR), to enable EH and information processing at the relay. Following that, Refs. [12,13] showed interest in the application of simultaneous wireless information and power transfer (SWIPT) for wireless communication systems. The authors in [12] studied the joint beamforming and power splitting design for a multi-user multiple-input single-output (MISO) broadcast system, where a multi-antenna base station (BS) simultaneously transmits information and power to a set of single-antenna mobile stations (MSs). Different from [12], Ref. [13] considered a large-scale network with multiple transmitter–receiver pairs where receivers conducted a PS technique to harvest energy from RF signals.

Most of the above works focused on EH using radio frequency (RF) transmitted from the source node. However, in practical communication networks, the RF signal is severely degraded due to the huge path loss between the source node and the receiver. Therefore, these systems are only suitable for short distance communications. To overcome this issue, Ref. [14] proposed a novel hybrid network with randomly deployed power beacons (PB) to provide a practically infinite battery lifetime for mobiles. PB-assisted wireless energy transfer has recently attracted a lot of attention from many researchers [15,16,17]. The authors of [15] analyzed the throughput of a distributed PB assisted wireless powered communication network via time division multiple access (TDMA) and under i.n.i.d. Nakagami-*m* fading distribution. The PB-assisted technique has been also studied in the device to device (D2D) communication system [16,17], due to the benefits of D2D systems, i.e., low latency, high spectral efficiency, and low transmit power [18]. In [19,20], multi-hop PB-assisted relaying schemes were studied and investigated. More specifically, the authors in [21,22] proposed novel multi-hop multi-path PB-assisted cooperating networks with path selection methods to enhance the system performance.

Besides energy, another consequence of the explosive growth of wireless services is the spectrum scarcity problem. To solve this problem, the concept of cognitive radio (CR) was first introduced by Mitola in [23], where licensed users (primary users (PUs)) can share their bands to unlicensed users (secondary users (SUs)) provided that quality of service (QoS) of the primary network is still guaranteed. Conventionally, SUs have to periodically sense the presence/absence of PUs, so that they can use vacant bands or move to another spectrum holes [24,25]. In [26,27], various spectrum sensing models for CR WSNs were introduced and compared. Refs. [26,27] also described the advantages of CR WSNs, the main difference between CR WSNs, conventional WSNs, and ad hoc CR networks. However, the transmission of SUs may be interrupted anytime due to the arrival of PUs, and this is the main disadvantage of the spectrum sensing methods. Recently, underlay CR protocols [28,29] were proposed to guarantee the continuous operation for SUs. In this method, SUs are allowed to utilize the licensed bands simultaneously with PUs provided that the secondary transmitters must adapt transmit power to satisfy an interference constraint given by PUs. To improve the performance of the secondary networks, cooperative relaying protocols [30,31,32,33] have been considered as a key technology, thanks to its capacity to increase the performances gains, i.e., coverage extension or transmission diversity, and power-saving transmission. In the literature, two proactive cooperative relaying strategies that have been widely investigated are opportunistic relay selection (ORS) [31,34], and partial relay selection (PRS) [35,36]. In ORS, the best relay is chosen to maximize the end-to-end (e2e) signal-to-noise ratio (SNR) between source and destination. In PRS, only the channel state information (CSI) of the source-relay links is used to select the relay for the cooperation. However, in [37], the authors proposed a new PRS scheme, where the relay is selected by using CSIs of the relay-destination links. In [37,38,39,40,41,42], different relay selection schemes in underlay CR networks were reported. Particularly, the authors in [37,38,39] evaluated the performance of the PRS protocols in terms of bit error rate (BER) and outage probability (OP). In [39,40,41], the cooperative cognitive schemes using the ORS methods were proposed and analyzed.

Naturally, the idea of EH and CR should be applied in WSNs to solve both the energy and spectrum scarcity issues. In [42], the authors considered the channel access problem utilizing Markov decision process (MDP), where SUs select a channel to access data transmission or harvest energy. Ref. [43] solved the optimization problem for the RF-EH-CR network with multiple SUs and multiple channels. Specifically, the authors proposed a system model in which SUs are able to harvest energy from a busy channel occupied by the primary user; the harvested energy is stored in the battery, and it is then used for data transmission over an idle channel. In order to tackle the energy efficiency and spectrum efficiency in CR, an EH-based DF two-way cognitive radio network (EH-TWCR) is proposed in [44]. In particular, the authors proposed two energy transfer policies, two relaying protocols, and two relay receiver structures to investigate the outage and throughput performance. In [45,46], the authors proposed the e2e performance of underlay multi-hop CR networks, where SUs can harvest energy from the power beacon [45] or from the RF signals of the primary transmitter [46].

Next, due to the low-cost transceiver hardware, sensor nodes are suffered from several kind of impairments such as phase noise, I/Q imbalance, amplifier nonlinearities, etc. [47,48,49]. To compensate the performance loss, cooperative relaying protocols can again be employed. Ref. [48] investigated the impact of hardware impairments on dual-hop relaying networks operating over Nakagami-*m* fading channels. In [49], outage probability and ergodic channel capacity of both PRS and ORS methods were measured under joint of co-channel interference and hardware impairments. In [50], the performance of two-way relaying schemes using EH relays with hardware imperfection in underlay CR networks was studied.

### 1.1. Motivations

In this paper, PB-assisted, hardware impairments, underlay cognitive radio, and cooperative relaying networks are combined into a novel cooperative spectrum sharing relaying system. Our proposed protocols not only improve the energy efficiency, but also the spectrum efficiency for the dual-hop decode-and-forward relaying WSNs. Different from multi-hop PB-assisted relaying schemes [19,20,21,22,45,46], this paper considers dual-hop PB-assisted cooperative networks with new relay selection methods. Firstly, we propose a hybrid PRS (H-PRS) protocol that combines the conventional PRS one in [13,36] and the modified one in [37]. Particularly, the scheme in [13,36] is used to select the cooperative relay if it obtains the lower value of OP; otherwise, the scheme in [37] is used. Secondly, to optimize the system performance, we propose a best ORS (B-ORS) protocol that outperforms the conventional ORS (C-ORS) one [34]. Finally, we attempt to evaluate the performance of the H-PRS, B-ORS and C-ORS protocols by providing closed-form expressions of the e2e OP and throughput (TP). The derived expressions are easy-to-compute, and hence they can be used to optimize the system performance.

### 1.2. Contributions

The main contributions of this paper can be summarized as follows:

- Three dual-hop DF cooperative relaying protocols are proposed. In H-PRS, the best relay can be selected by using the CSIs of the first or second hop. On the other hand, C-ORS and B-ORS select a relay that has the highest e2e channel gain and the highest e2e SNRs, respectively, to convey the data transmission from secondary source to secondary destination.
- It is noteworthy that the PB-assisted cooperative CR relaying systems using H-PRS, B-ORS, or C-ORS have their own mathematical analysis challenges since the energy harvested from the beacon and the interference constraint of the primary users (PUs) affect the transmit power of the secondary source and relays. Moreover, due to the correlation between SNRs of the first and second hop, the analysis of the performance in the C-ORS scheme becomes much more challenging, compared with that in the H-PRS and B-ORS schemes.
- Assuming independently and identically distributed (i.i.d.) Rayleigh fading environment, exact closed-form expressions and asymptotic analysis of OP and TP for H-PRS, B-ORS and C-ORS are derived. Monte Carlo simulations are performed to validate our derivations.

The rest of paper is organized as follows. Section 2 describes the system model used in this paper. Section 3 provides the performance evaluation. Section 4 gives the simulation results while Section 5 concludes the paper.

## 2. System Model

**Figure 1:**
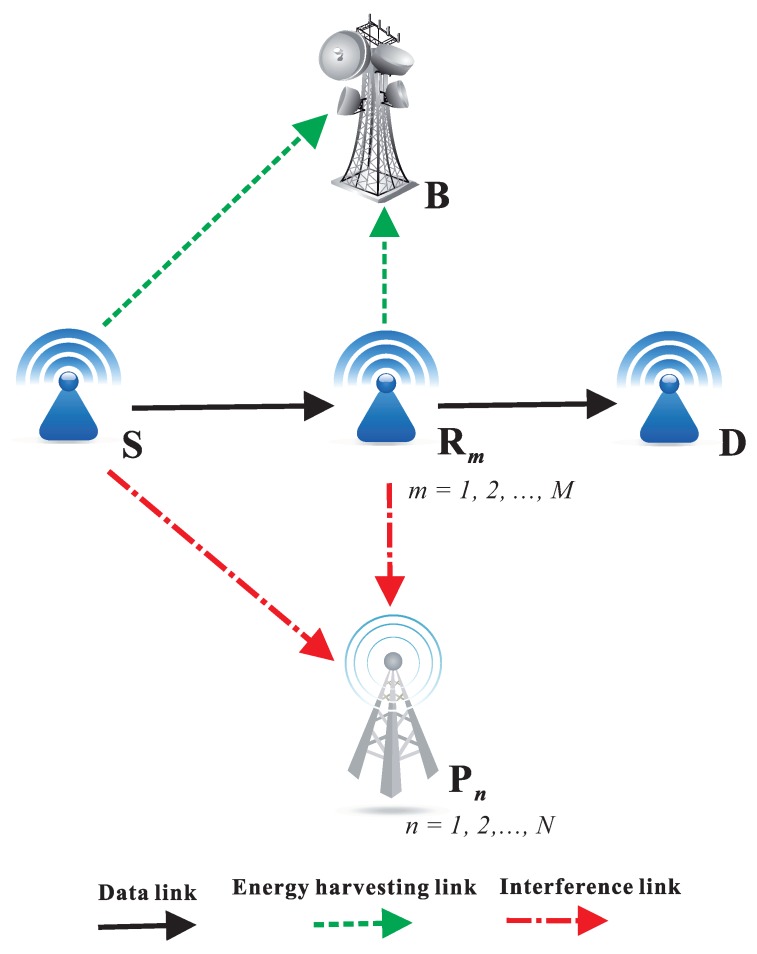
System model of PB-assisted relaying protocols in underlay CR with relay selection methods.

Figure 1 presents the system model of the proposed CR WSNs. In the secondary network, a source S communicates with a destination D in the dual-hop fashion. In addition, there are *M* secondary relays (denoted by $R_{1},\;R_{2},\ldots ,R_{M}\;$), and one of them is selected to serve the source-destination communication. In the primary network, there are *N* licensed users (or primary users), denoted as $P_{1},\;P_{2},\ldots ,P_{N}\;$. To support dynamic spectrum access in a strict manner, the secondary transmitters must adjust their transmit power so that the interferences generated by their operations are not harmful to the quality of service (QoS) of the primary users. It is assumed that the source and relays are single-antenna and energy-constrained devices that have to harvest energy from a *K*-antenna power beacon (B) deployed in the secondary network. Due to deep shadow fading or far distance, the direct link between S and D does not exist, and the data transmission is realized by two orthogonal time slots via the selected relay.

Denote $\gamma_{SR_{m}}={\left|h_{SR_{m}}\right|}^{2}$ and $\gamma_{R_{m}D}={\left|h_{R_{m}D}\right|}^{2}$ as the channel gains of the $S\to R_{m}$ and $R_{m}\to D$ links, respectively, where m=1,2,…,M. For the interference links, $\gamma_{SP_{n}}$ and $\gamma_{R_{m}P_{n}}$ denote the channel gains of the $S\to P_{n}$ and $R_{m}\to P_{n}$ links, where n=1,2,…,N. Next, the channel gains between the *k*-th antenna of the beacon and the source and relay $R_{m}$ are $\gamma_{B_{k}S}$ and $\gamma_{B_{k}R_{m}}$, respectively, where k=1,2,…,K. Assume that all of the channels experience Rayleigh fading, and hence the channel gains have exponential distributions. Denote $\lambda_{\mathrm{XY}}$ as a parameter of the random variable (RV) $\gamma_{\mathrm{XY}}$, which is given as $\lambda_{\mathrm{XY}}=1/E\left\{\gamma_{\mathrm{XY}}\right\}$, where $\left(X,Y\right)\in \left\{S,R_{m},D,B_{k},P_{n}\right\}$, and $E\left\{Z\right\}$ is the expected value of a RV *Z*. Therefore, the cumulative distribution function (CDF) and probability density function (PDF) of the RV $\gamma_{\mathrm{XY}}$ can be expressed, respectively, as

(1) $$\begin{array}{c} F_{\gamma_{\mathrm{XY}}}\left(x\right)=1-\exp\left(-\lambda_{\mathrm{XY}}x\right),\;\;f_{\gamma_{\mathrm{XY}}}\left(x\right)=\lambda_{\mathrm{XY}}\exp\left(-\lambda_{\mathrm{XY}}x\right). \end{array}$$

To take path-loss into account, we can model these parameters as in [30]:

(2) $$\begin{array}{c} \lambda_{\mathrm{XY}}=d_{\mathrm{XY}}^{\beta }, \end{array}$$

where β is the path-loss exponent, and $d_{\mathrm{XY}}$ is the link distance between the nodes X and Y.

Assume that the relays (and primary uses) are close together and form a cluster. Hence, $d_{SR_{m}}=d_{\mathrm{SR}},$ $d_{SP_{n}}=d_{\mathrm{SP}}$ and $d_{R_{m}P_{n}}=d_{\mathrm{RP}}$ can be assumed for all *m* and *n*. Hence, $\gamma_{SR_{m}}$ (and $\gamma_{R_{m}D},\;\gamma_{SP_{n}},\;\gamma_{R_{m}P_{n}}$) are i.i.d. RVs can be assumed, where $\lambda_{SR_{m}}=\lambda_{\mathrm{SR}},\;\lambda_{R_{m}D}=\lambda_{\mathrm{RD}},\;\lambda_{SP_{n}}=\lambda_{\mathrm{SP}}$ and $\lambda_{R_{m}P_{n}}=\lambda_{\mathrm{RP}}$ for all *m* and *n*. Similarly, $\gamma_{B_{k}S}$ and $\gamma_{B_{k}R_{m}}$ are also assumed to be i.i.d. RVs, i.e., $\lambda_{B_{k}S}=\lambda_{\mathrm{BS}}$ and $\lambda_{B_{k}R_{m}}=\lambda_{\mathrm{BR}}$ for all *k* and *m*.

Next, denote T as the duration of each data transmission from the source to the destination. By using the TSR protocol [11], a duration of αT is used for the EH process, while the time spent for both the S-R and R-D transmission is $\left(1-\alpha \right)T/2$, where 0≤α≤1.

### 2.1. Hardware Impairments

In the presence of hardware impairments, the received signal of the transmission X→Y link can be expressed as

(3) $$\begin{array}{c} y_{\mathrm{XY}}=\sqrt{P_{X}}h_{\mathrm{XY}}\left(s+\eta_{\mathrm{XY}}\right)+\mu_{\mathrm{XY}}+\nu_{\mathrm{XY}}, \end{array}$$

where $P_{X}$ denotes the transmit power of the transmitter X, $h_{\mathrm{XY}}$ is the channel coefficient of the X→Y link, $\eta_{\mathrm{XY}}$ and $\mu_{\mathrm{XY}}$ denotes noises caused by the hardware impairments at the transmitter X and the receiver Y, respectively, and $\nu_{\mathrm{XY}}$ are the additive white Gaussian noises models as Gaussian random variables with zero mean and variance $N_{0}$.

**Remark** **1.** *Similar to [48,49,50], we can model the distortion noises $\eta_{\mathrm{XY}}$ and $\mu_{\mathrm{XY}}$ as circularly-symmetric complex Gaussian distribution with zero-mean and variance $\tau_{X}^{2}P_{X}$ and $\tau_{Y}^{2}P_{X}\gamma_{\mathrm{XY}},$ respectively.*

Let us consider the communication between the transmitter X and the receiver Y, and the obtained instantaneous SNR of the X-Y link can be formulated by (see [48,49,50])

(4) $$\begin{array}{c} \Psi =\frac{P_{X}\gamma_{\mathrm{XY}}}{\left(\tau_{X}^{2}+\tau_{Y}^{2}\right)P_{X}\gamma_{\mathrm{XY}}+N_{0}}=\frac{P_{X}\gamma_{\mathrm{XY}}}{\tau_{\mathrm{XY}}^{2}P_{X}\gamma_{\mathrm{XY}}+N_{0}}, \end{array}$$

where $\tau_{X}^{2}$ and $\tau_{Y}^{2}$ present the levels of the hardware impairments at the transmitter X and the receiver Y, respectively, $\tau_{\mathrm{XY}}^{2}=\tau_{X}^{2}+\tau_{Y}^{2}$ is defined as the total hardware impairment level of the X-Y link, and $N_{0}$ is the variance of Gaussian noise at Y.

For ease of presentation and analysis, the impairment levels of the data links and interference links are assumed that $\tau_{SR_{m}}^{2}=\tau_{R_{m}D}^{2}=\tau_{D}^{2}$ for all *m*, and $\tau_{SP_{n}}^{2}=\tau_{R_{m}P_{n}}^{2}=\tau_{I}^{2}$ for all *m* and *n*.

### 2.2. Energy Harvesting Phase

In this phase, node B uses all of the antennas to support the energy for the source and the relays. Then, the energy harvested by the source and the relay $R_{m}$ can be given, respectively, by (see [15])

(5) $$\begin{array}{c} Q_{S}=\eta \alpha TP_{B}\sum_{k=1}^{K}\gamma_{B_{k}S}, \end{array}$$

(6) $$\begin{array}{c} Q_{R_{m}}=\eta \alpha TP_{B}\sum_{k=1}^{K}\gamma_{B_{k}R_{m}}, \end{array}$$

where $P_{B}$ is the transmit power of B, and η is the energy conversion efficiency at S and $R_{m}$, αT is time used for the EH process, $\sum_{k=1}^{K}\gamma_{B_{k}S}$ and $\sum_{k=1}^{K}\gamma_{B_{k}R_{m}}$ are channel gains of the EH links, i.e., $B_{k}\to S$ and $B_{k}\to R_{m}$ links, respectively.

From Equations (5) and (6), the average transmit power that the nodes S and $R_{m}$ can utilize is formulated, respectively, by

(7) $$\begin{array}{c} E_{S}=\frac{Q_{S}}{\left(1-\alpha \right)T/2}=\kappa P_{B}X_{0}^{\mathrm{sum}}, \end{array}$$

(8) $$\begin{array}{c} E_{R_{m}}=\frac{Q_{R_{m}}}{\left(1-\alpha \right)T/2}=\kappa P_{B}X_{m}^{\mathrm{sum}}, \end{array}$$

where

(9) $$\begin{array}{c} \kappa =\frac{2\eta \alpha }{1-\alpha },X_{0}^{\mathrm{sum}}\;=\sum_{k=1}^{K}\gamma_{B_{k}S},X_{m}^{\mathrm{sum}}=\sum_{k=1}^{K}\gamma_{B_{k}R_{m}}. \end{array}$$

### 2.3. Transmit Power Formulation

In underlay CR, the nodes S and $R_{m}$ must adjust their transmit power to satisfy the interference constraint (see [39]), i.e.,

(10) $$\begin{array}{c} I_{S}\leq \frac{I_{\mathrm{th}}}{\left(1+\tau_{I}^{2}\right)\max_{n=1,2,\ldots ,N}\left(\gamma_{SP_{n}}\right)}=\frac{I_{\mathrm{th}}}{\left(1+\tau_{I}^{2}\right)Y_{0}^{\max}}, \end{array}$$

(11) $$\begin{array}{c} I_{R_{m}}\leq \frac{I_{\mathrm{th}}}{\left(1+\tau_{I}^{2}\right)\max_{n=1,2,\ldots ,N}\left(\gamma_{R_{m}P_{n}}\right)}=\frac{I_{\mathrm{th}}}{\left(1+\tau_{I}^{2}\right)Y_{m}^{\max}}, \end{array}$$

where $I_{\mathrm{th}}$ is the interference constraint threshold required by the primary users, and:

(12) $$\begin{array}{c} Y_{0}^{\max}=\max_{n=1,2,\ldots ,N}\left(\gamma_{SP_{n}}\right),\;Y_{m}^{\max}=\max_{n=1,2,\ldots ,N}\left(\gamma_{R_{m}P_{n}}\right). \end{array}$$

From Equations (7)–(8), and (10)–(11), the maximum transmit power of S and $R_{m}$ can be formulated, respectively, as

(13) $$\begin{array}{c} P_{0}=\min\left(E_{S},I_{S}\right)=P_{B}\min\left(\kappa X_{0}^{\mathrm{sum}},\frac{\delta }{Y_{0}^{\max}}\right), \end{array}$$

(14) $$\begin{array}{c} P_{m}=\min\left(E_{R_{m}},I_{R_{m}}\right)=P_{B}\min\left(\kappa X_{m}^{\mathrm{sum}},\frac{\delta }{Y_{m}^{\max}}\right), \end{array}$$

where $\delta =I_{\mathrm{th}}/P_{B}/\left(1+\tau_{I}^{2}\right).$ In addition, we denote $\mu =I_{\mathrm{th}}/P_{B}$ that is assumed to be a constant.

Then, under the impact of the hardware impairments, the instantaneous SNR obtained at the first and second hops across the relay can be given, respectively, by

(15) $$\begin{array}{c} \Psi_{1m}=\frac{P_{0}\gamma_{SR_{m}}}{\tau_{D}^{2}P_{0}\gamma_{SR_{m}}+N_{0}}=\frac{\Delta \min\left(\kappa X_{0}^{\mathrm{sum}},\delta /Y_{0}^{\max}\right)\gamma_{SR_{m}}}{\tau_{D}^{2}\Delta \min\left(\kappa X_{0}^{\mathrm{sum}},\delta /Y_{0}^{\max}\right)\gamma_{SR_{m}}+1}, \end{array}$$

(16) $$\begin{array}{c} \Psi_{2m}=\frac{P_{m}\gamma_{R_{m}D}}{\tau_{D}^{2}P_{m}\gamma_{R_{m}D}+N_{0}}=\frac{\Delta \min\left(\kappa X_{m}^{\mathrm{sum}},\delta /Y_{m}^{\max}\right)\gamma_{R_{m}D}}{\tau_{D}^{2}\Delta \min\left(\kappa X_{m}^{\mathrm{sum}},\delta /Y_{m}^{\max}\right)\gamma_{R_{m}D}+1}, \end{array}$$

where $\Delta =P_{B}/N_{0},$ $N_{0}$ is the additive white Gaussian noise (AWGN) variance.

With the DF relaying technique, the e2e channel capacity of the $S\to R_{m}\to D$ path is formulated by

(17) $$\begin{array}{c} C_{m}=\frac{\left(1-\alpha \right)T}{2}\log_{2}\left(1+\min\left(\Psi_{1m},\Psi_{2m}\right)\right). \end{array}$$

From (17), the e2e outage probability is defined as the probability that the end-to-end capacity is lower than a positive threshold, i.e., $C_{\mathrm{th}}$ as follows:

(18) $$\begin{array}{c} \mathrm{OP}=\mathrm{Pr}\left(C_{m}<C_{\mathrm{th}}\right), \end{array}$$

where $C_{\mathrm{th}}$ is the target data rate of the secondary network.

Then, the e2e throughput (TP) can be formulated as in [11]:

(19) $$\begin{array}{c} \mathrm{TP}=\left(1-\alpha \right)TC_{\mathrm{th}}\left(1-\mathrm{OP}\right), \end{array}$$

where $\left(1-\alpha \right)T$ is the total transmission time, i.e., $S\to R_{m}\to D.$

### 2.4. Relay Selection Methods

#### 2.4.1. Hybrid Partial Relay Selection (H-PRS)

In the conventional PRS protocol [35], the relay providing the highest channel gain at the first hop is selected to forward the data to the destination. Mathematically speaking, we write

(20) $$\begin{array}{c} R_{a_{1}}:\gamma_{SR_{a_{1}}}=\max_{m=1,2,\ldots ,M}\left(\gamma_{SR_{m}}\right), \end{array}$$

where $R_{a_{1}}$ is the chosen relay with $a_{1}\in \left\{1,2,\ldots ,M\right\}.$

For the PRS protocol proposed in [37], the best relay is selected by the following strategy:

(21) $$\begin{array}{c} R_{a_{2}}:\gamma_{R_{a_{2}}D}=\max_{m=1,2,\ldots ,M}\left(\gamma_{R_{m}D}\right), \end{array}$$

where $a_{2}\in \left\{1,2,\ldots ,M\right\}.$

Combining Equations (17) and (18) and Equations (20) and (21), the e2e OP of the PRS methods in [35,37] can be expressed, respectively, as

(22) $$\begin{array}{c} OP_{\mathrm{PRS}1}=\mathrm{Pr}\left(C_{a_{1}}<C_{\mathrm{th}}\right)=\mathrm{Pr}\left(\frac{\left(1-\alpha \right)T}{2}\log_{2}\left(1+\min\left(\Psi_{1a_{1}},\Psi_{2a_{1}}\right)\right)<C_{\mathrm{th}}\right), \end{array}$$

(23) $$\begin{array}{c} OP_{\mathrm{PRS}2}=\mathrm{Pr}\left(C_{a_{2}}<C_{\mathrm{th}}\right)=\mathrm{Pr}\left(\frac{\left(1-\alpha \right)T}{2}\log_{2}\left(1+\min\left(\Psi_{1a_{2}},\Psi_{2a_{2}}\right)\right)<C_{\mathrm{th}}\right). \end{array}$$

In our proposed PRS protocol, if $OP_{\mathrm{PRS}1}\leq OP_{\mathrm{PRS}2}$, the best relay is selected by (20), and if $OP_{\mathrm{PRS}1}>OP_{\mathrm{PRS}2}$, the selection method in (21) is used to choose the relay for the cooperation (the operation of the H-PRS protocol will be described in the next sections). As a result, the outage performance of the H-PRS protocol is expressed as

(24) $$\begin{array}{c} OP_{H-\mathrm{PRS}}=\min\left(OP_{\mathrm{PRS}1},OP_{\mathrm{PRS}2}\right). \end{array}$$

Next, the obtained throughput of this protocol is calculated by

(25) $$\begin{array}{c} TP_{H-\mathrm{PRS}}=\left(1-\alpha \right)TC_{\mathrm{th}}\left(1-OP_{H-\mathrm{PRS}}\right). \end{array}$$

#### 2.4.2. Best Opportunistic Relay Selection (B-ORS)

In the B-ORS protocol, the best relay is chosen to maximize the e2e SNR, i.e.,

(26) $$\begin{array}{c} R_{b:}\min\left(\Psi_{1b},\Psi_{2b}\right)=\max_{m=1,2,\ldots ,M}\left(\min\left(\Psi_{1m},\Psi_{2m}\right)\right), \end{array}$$

where $b\in \left\{1,2,\ldots ,M\right\}.$

Then, the e2e performances of this scheme are given, respectively, by

(27) $$\begin{array}{cc} OP_{B-\mathrm{ORS}} & =\mathrm{Pr}\left(\frac{\left(1-\alpha \right)T}{2}\log_{2}\left(1+\min\left(\Psi_{1b},\Psi_{2b}\right)\right)<C_{\mathrm{th}}\right),\\ TP_{B-\mathrm{ORS}} & =\left(1-\alpha \right)TC_{\mathrm{th}}\left(1-OP_{B-\mathrm{ORS}}\right). \end{array}$$

#### 2.4.3. Conventional Opportunistic Relay Selection (C-ORS)

As proposed in much of the literature such as [31,34,40,49], the best relay is selected to maximize the end-to-end SNR of the data link:

(28) $$\begin{array}{c} R_{c:}\min\left(\gamma_{SR_{c}},\gamma_{R_{c}D}\right)=\max_{m=1,2,\ldots ,M}\left(\min\left(\gamma_{SR_{m}},\gamma_{R_{m}D}\right)\right), \end{array}$$

where $c\in \left\{1,2,\ldots ,M\right\}.$

Then, the e2e OP and e2e TP of the C-ORS protocol is computed as

(29) $$\begin{array}{cc} OP_{C-\mathrm{ORS}} & =\mathrm{Pr}\left(\frac{\left(1-\alpha \right)T}{2}\log_{2}\left(1+\min\left(\Psi_{1c},\Psi_{2c}\right)\right)<C_{\mathrm{th}}\right),\\ TP_{C-\mathrm{ORS}} & =\left(1-\alpha \right)TC_{\mathrm{th}}\left(1-OP_{C-\mathrm{ORS}}\right). \end{array}$$

It is worth noting that the implementation of C-ORS is simpler than that of B-ORS because it only requires perfect CSIs of the data links.

## 3. Performance Evaluation

### 3.1. Outage Probability

Generally, the e2e OP of the protocol U, $U\in \left\{H-\mathrm{PRS},B-\mathrm{ORS},C-\mathrm{ORS}\right\},$ can be expressed as follows:

(30) $$\begin{array}{cc} OP_{U} & =\mathrm{Pr}\left(\min\left(\Psi_{1l},\Psi_{2l}\right)<\theta \right)=1-\mathrm{Pr}\left(\min\left(\Psi_{1l},\Psi_{2l}\right)\geq \theta \right)\\ & =1-\mathrm{Pr}\left(\Psi_{1l}\geq \theta ,\Psi_{2l}\geq \theta \right), \end{array}$$

where $l\in \left\{a_{1},a_{2},b,c\right\}$ and

(31) $$\begin{array}{c} \theta =2^{\frac{2C_{\mathrm{th}}}{\left(1-\alpha \right)T}}-1. \end{array}$$

Moreover, substituting (15) and (16) into (30), yields

(32) $$\begin{array}{c} OP_{U}=1- \end{array}\mathrm{Pr}\left(\left(1-\tau_{D}^{2}\theta \right)\Delta \min\left(\kappa X_{0}^{\mathrm{sum}},\frac{\delta }{Y_{0}^{\max}}\right)\gamma_{SR_{l}}\geq \theta ,\left(1-\tau_{D}^{2}\theta \right)\Delta \min\left(\kappa X_{l}^{\mathrm{sum}},\frac{\delta }{Y_{l}^{\max}}\right)\gamma_{R_{l}D}\geq \theta \right).$$

It is obvious from (32) that $OP_{U}=1,$ if $1-\tau_{D}^{2}\theta \leq 0$. In the case that $1-\tau_{D}^{2}\theta >0$, Equation (32) can be expressed under the following form:

(33) $$\begin{array}{c} OP_{U}=1-\mathrm{Pr}\left(\min\left(\kappa X_{0}^{\mathrm{sum}},\frac{\delta }{Y_{0}^{\max}}\right)\gamma_{SR_{l}}\geq \frac{\rho }{\Delta },\min\left(\kappa X_{l}^{\mathrm{sum}},\frac{\delta }{Y_{l}^{\max}}\right)\gamma_{R_{l}D}\geq \frac{\rho }{\Delta }\right), \end{array}$$

where $\rho =\theta /\left(1-\tau_{D}^{2}\theta \right).$

**Lemma** **1.** *As $1-\tau_{D}^{2}\theta >0$, exact closed-form expressions of $OP_{\mathrm{PRS}1}$ and $OP_{\mathrm{PRS}2}$ can be given, respectively, as*

(34) $$\begin{array}{c} \begin{array}{cc} OP_{\mathrm{PRS}1}=1- & \left[\begin{array}{c} \sum_{t=0}^{K-1}{\sum_{m=0}^{M-1}{\left(-1\right)}^{m}\frac{2C_{M-1}^{m}M}{t!}{\left(m+1\right)}^{\frac{t-1}{2}}\left(\frac{\lambda_{\mathrm{BS}}\lambda_{\mathrm{SR}}\rho }{\kappa \Delta }\right)}^{\frac{t+1}{2}}K_{1-t}\left(2\sqrt{\frac{\left(m+1\right)\lambda_{\mathrm{BS}}\lambda_{\mathrm{SR}}\rho }{\kappa \Delta }}\right)\\ -\sum_{t=0}^{K-1}{\sum_{n=1}^{N}\sum_{m=0}^{M-1}{\left(-1\right)}^{n+m+1}C_{M-1}^{m}C_{N}^{n}\frac{2M\lambda_{\mathrm{SR}}}{t!}{\left(\frac{\rho }{n\lambda_{\mathrm{SP}}\delta \Delta +\left(m+1\right)\lambda_{\mathrm{SR}}\rho }\right)}^{\frac{1-t}{2}}\left(\frac{\lambda_{\mathrm{BS}}\rho }{\kappa \Delta }\right)}^{\frac{t+1}{2}}\\ \times K_{1-t}\left(2\sqrt{\frac{\lambda_{\mathrm{BS}}}{\kappa \Delta }\left(n\lambda_{\mathrm{SP}}\delta \Delta +\left(m+1\right)\lambda_{\mathrm{SR}}\rho \right)}\right) \end{array}\right]\\ \times & \left[\begin{array}{c} \sum_{t=0}^{K-1}\frac{2}{t!}{\left(\frac{\lambda_{\mathrm{BR}}\lambda_{\mathrm{RD}}\rho }{\kappa \Delta }\right)}^{\frac{t+1}{2}}K_{1-t}\left(2\sqrt{\frac{\lambda_{\mathrm{BR}}\lambda_{\mathrm{RD}}\rho }{\kappa \Delta }}\right)\\ -\sum_{t=0}^{K-1}\sum_{n=1}^{N}{\left(-1\right)}^{n+1}C_{N}^{n}\frac{2\lambda_{\mathrm{RD}}}{t!}{\left(\frac{\rho }{n\lambda_{\mathrm{RP}}\delta \Delta +\lambda_{\mathrm{RD}}\rho }\right)}^{\frac{1-t}{2}}{\left(\frac{\lambda_{\mathrm{BR}}\rho }{\kappa \Delta }\right)}^{\frac{t+1}{2}}\\ \times K_{1-t}\left(2\sqrt{\frac{\lambda_{\mathrm{BR}}}{\kappa \Delta }\left(n\lambda_{\mathrm{RP}}\delta \Delta +\lambda_{\mathrm{RD}}\rho \right)}\right) \end{array}\right], \end{array} \end{array}$$

(35) $$\begin{array}{c} \begin{array}{cc} OP_{\mathrm{PRS}2}=1- & \left[\begin{array}{c} \sum_{t=0}^{K-1}{\sum_{m=0}^{M-1}{\left(-1\right)}^{m}\frac{2C_{M-1}^{m}M}{t!}{\left(m+1\right)}^{\frac{t-1}{2}}\left(\frac{\lambda_{\mathrm{BR}}\lambda_{\mathrm{RD}}\rho }{\kappa \Delta }\right)}^{\frac{t+1}{2}}K_{1-t}\left(2\sqrt{\frac{\left(m+1\right)\lambda_{\mathrm{BR}}\lambda_{\mathrm{RD}}\rho }{\kappa \Delta }}\right)\\ -\sum_{t=0}^{K-1}{\sum_{n=1}^{N}\sum_{m=0}^{M-1}{\left(-1\right)}^{n+m+1}C_{M-1}^{m}C_{N}^{n}\frac{2M\lambda_{\mathrm{RD}}}{t!}{\left(\frac{\rho }{n\lambda_{\mathrm{RP}}\delta \Delta +\left(m+1\right)\lambda_{\mathrm{RD}}\rho }\right)}^{\frac{1-t}{2}}\left(\frac{\lambda_{\mathrm{BR}}\rho }{\kappa \Delta }\right)}^{\frac{t+1}{2}}\\ \times K_{1-t}\left(2\sqrt{\frac{\lambda_{\mathrm{BR}}}{\kappa \Delta }\left(n\lambda_{\mathrm{RP}}\delta \Delta +\left(m+1\right)\lambda_{\mathrm{RD}}\rho \right)}\right) \end{array}\right]\\ \times & \left[\begin{array}{c} \sum_{t=0}^{K-1}\frac{2}{t!}{\left(\frac{\lambda_{\mathrm{BS}}\lambda_{\mathrm{SR}}\rho }{\kappa \Delta }\right)}^{\frac{t+1}{2}}K_{1-t}\left(2\sqrt{\frac{\lambda_{\mathrm{BS}}\lambda_{\mathrm{SR}}\rho }{\kappa \Delta }}\right)\\ -\sum_{t=0}^{K-1}\sum_{n=1}^{N}{\left(-1\right)}^{n+1}C_{N}^{n}\frac{2\lambda_{\mathrm{SR}}}{t!}{\left(\frac{\rho }{n\lambda_{\mathrm{SP}}\delta \Delta +\lambda_{\mathrm{SR}}\rho }\right)}^{\frac{1-t}{2}}{\left(\frac{\lambda_{\mathrm{BS}}\rho }{\kappa \Delta }\right)}^{\frac{t+1}{2}}\\ \times K_{1-t}\left(2\sqrt{\frac{\lambda_{\mathrm{BS}}}{\kappa \Delta }\left(n\lambda_{\mathrm{SP}}\delta \Delta +\lambda_{\mathrm{SR}}\rho \right)}\right) \end{array}\right]. \end{array} \end{array}$$

As mentioned in (24), we have $OP_{H-\mathrm{PRS}}=\min\left(OP_{\mathrm{PRS}1},OP_{\mathrm{PRS}2}\right).$ In addition, the operation of the H-PRS protocol can be realized as follows. At first, we assume that the source (S) and the destination (D) can know the statistical information of the data links (i.e., $\lambda_{\mathrm{SR}},\lambda_{\mathrm{RD}}$), the interference links (i.e., $\lambda_{\mathrm{SP}},\lambda_{\mathrm{RP}}$) and the EH links (i.e., $\lambda_{\mathrm{BS}},\lambda_{\mathrm{BR}}$). In practice, the statistical CSIs can be easily obtained by averaging the instantaneous CSI [51,52], and they can be known by all of the nodes via control messages. Next, the source and destination nodes can calculate $OP_{\mathrm{PRS}1},OP_{\mathrm{PRS}2}$ by using (34) and (35), respectively. Finally, by comparing $OP_{\mathrm{PRS}1}$ and $OP_{\mathrm{PRS}2}$, the source (or the destination) can decide to use the scheme in [35] or in [37] for the source-destination data transmission.

**Proof.**  Firstly, we calculate the outage probability $OP_{\mathrm{PRS}1}$. Due to the independence between $\Psi_{1a_{1}}$ and $\Psi_{1a_{2}}$, we can rewrite (33) as

(36) $$\begin{array}{cc} OP_{\mathrm{PRS}1} & =1-\mathrm{Pr}\left(\min\left(\kappa X_{0}^{\mathrm{sum}},\frac{\delta }{Y_{0}^{\max}}\right)\gamma_{SR_{a_{1}}}\geq \frac{\rho }{\Delta }\right)\mathrm{Pr}\left(\min\left(\kappa X_{a_{1}}^{\mathrm{sum}},\frac{\delta }{Y_{a_{1}}^{\max}}\right)\gamma_{R_{a_{1}}D}\geq \frac{\rho }{\Delta }\right)\\ & =1-\left(1-A_{1}\right)\left(1-A_{2}\right), \end{array}$$

where $A_{1}$ and $A_{2}$ are outage probability at the first and second hops, respectively, given as

(37) $$\begin{array}{cc} A_{1} & =\mathrm{Pr}\left(\min\left(\kappa X_{0}^{\mathrm{sum}},\frac{\delta }{Y_{0}^{\max}}\right)\gamma_{SR_{a_{1}}}<\frac{\rho }{\Delta }\right),\\ A_{2} & =\mathrm{Pr}\left(\min\left(\kappa X_{a_{1}}^{\mathrm{sum}},\frac{\delta }{Y_{a_{1}}^{\max}}\right)\gamma_{R_{a_{1}}D}<\frac{\rho }{\Delta }\right). \end{array}$$

Next, we can rewrite $A_{1}$ as

(38) $$\begin{array}{c} A_{1}=\int_{0}^{+\infty }F_{Z_{1}}\left(\frac{\rho }{\Delta x}\right)f_{\gamma_{SR_{a_{1}}}}\left(x\right)dx, \end{array}$$

where $Z_{1}=\min\left(\kappa X_{0}^{\mathrm{sum}},\frac{\delta }{Y_{0}^{\max}}\right)$, $F_{Z_{1}}\left(.\right)$ and $f_{\gamma_{SR_{a_{1}}}}\left(.\right)$ are CDF and PDF of $Z_{1}$ and $\gamma_{SR_{a_{1}}}$, respectively.

Combining Equations (1) and (20), $F_{\gamma_{SR_{a_{1}}}}\left(.\right)$ can be given as

(39) $$\begin{array}{cc} F_{\gamma_{SR_{a_{1}}}}\left(x\right) & =\mathrm{Pr}\left(\max_{m=1,2,\ldots ,M}\left(\gamma_{SR_{m}}\right)<x\right)={\left(F_{\gamma_{SR_{m}}}\left(x\right)\right)}^{M}\\ & ={\left(1-\exp\left(-\lambda_{\mathrm{SR}}x\right)\right)}^{M}. \end{array}$$

From (39), the corresponding PDF can be obtained as

(40) $$\begin{array}{cc} f_{\gamma_{SR_{a}}}\left(x\right)\; & =M\lambda_{\mathrm{SR}}\exp\left(-\lambda_{\mathrm{SR}}x\right){\left(1-\exp\left(-\lambda_{\mathrm{SR}}x\right)\right)}^{M-1}\\ & =\sum_{m=0}^{M-1}{\left(-1\right)}^{m}C_{M-1}^{m}M\lambda_{\mathrm{SR}}\exp\left(-\left(m+1\right)\lambda_{\mathrm{SR}}x\right), \end{array}$$

where $C_{M-1}^{m}$ is a binomial coefficient.

Considering the RV $Z_{1},$ its CDF can be formulated by

(41) $$\begin{array}{cc} F_{Z_{1}}\left(x\right) & =\mathrm{Pr}\left(\min\left(\kappa X_{0}^{\mathrm{sum}},\frac{\delta }{Y_{0}^{\max}}\right)<x\right)\\ & =1-\left(1-F_{X_{0}^{\mathrm{sum}}}\left(\frac{x}{\kappa }\right)\right)F_{Y_{0}^{\max}}\left(\frac{\delta }{x}\right). \end{array}$$

From (9), since $X_{0}^{\mathrm{sum}}$ is the sum of i.i.d. exponential RVs, CDF $F_{X_{0}^{\mathrm{sum}}}\left(x/\kappa \right)$ in (41) can be given as

(42) $$\begin{array}{c} F_{X_{0}^{\mathrm{sum}}}\left(\frac{x}{\kappa }\right)=1-\exp\left(\frac{\lambda_{\mathrm{BS}}}{\kappa }x\right)\sum_{t=0}^{K-1}{\frac{1}{t!}\left(\frac{\lambda_{\mathrm{BS}}}{\kappa }\right)}^{t}x^{t}. \end{array}$$

Next, from (12), we can obtain CDF $F_{Y_{0}^{\max}}\left(\delta /x\right)$, similar to (39), as

(43) $$\begin{array}{c} F_{Y_{0}^{\max}}\left(\frac{\delta }{x}\right)=1+\sum_{n=1}^{N}{\left(-1\right)}^{n}C_{N}^{n}\exp\left(-\frac{n\lambda_{\mathrm{SP}}\delta }{x}\right). \end{array}$$

Substituting (42) and (43) into (41), we have

(44) $$\begin{array}{cc} F_{Z_{1}}\left(x\right)=1 & -\sum_{t=0}^{K-1}{\frac{1}{t!}\left(\frac{\lambda_{\mathrm{BS}}}{\kappa }\right)}^{t}x^{t}\exp\left(-\frac{\lambda_{\mathrm{BS}}}{\kappa }x\right)\\ & -\sum_{t=0}^{K-1}{\sum_{n=1}^{N}{\left(-1\right)}^{n}\frac{C_{N}^{n}}{t!}\left(\frac{\lambda_{\mathrm{BS}}}{\kappa }\right)}^{t}x^{t}\exp\left(-\frac{\lambda_{\mathrm{BS}}}{\kappa }x-\frac{n\lambda_{\mathrm{SP}}\delta }{x}\right). \end{array}$$

Then, substituting (40) and (44) into (38), we arrive at

(45) $$\begin{array}{cc} A_{1} & =1-\sum_{t=0}^{K-1}{\sum_{m=0}^{M-1}{\left(-1\right)}^{m}C_{M-1}^{m}\frac{M\lambda_{\mathrm{SR}}}{t!}\left(\frac{\lambda_{\mathrm{BS}}\rho }{\kappa \Delta }\right)}^{t}\int_{0}^{+\infty }\frac{1}{x^{t}}\exp\left(-\frac{\lambda_{\mathrm{BS}}\rho }{\kappa \Delta }\frac{1}{x}\right)\exp\left(-\left(m+1\right)\lambda_{\mathrm{SR}}x\right)dx\\ & -\sum_{t=0}^{K-1}{\sum_{n=1}^{N}\sum_{m=0}^{M-1}{\left(-1\right)}^{n+m}C_{N}^{n}C_{M-1}^{m}\frac{M\lambda_{\mathrm{SR}}}{t!}\left(\frac{\lambda_{\mathrm{BS}}\rho }{\kappa \Delta }\right)}^{t}\int_{0}^{+\infty }\frac{1}{x^{t}}\exp\left(-\frac{\lambda_{\mathrm{BS}}\rho }{\kappa \Delta }\frac{1}{x}\right)\\ & \times \exp\left(-\left(\frac{n\lambda_{\mathrm{SP}}\delta \Delta }{\rho }+\left(m+1\right)\lambda_{\mathrm{SR}}\right)x\right)dx. \end{array}$$

Using ([53], Equation (3.471.9)) for the corresponding integrals in (45), we obtain

(46) $$\begin{array}{cc} A_{1} & =1-\sum_{t=0}^{K-1}{\sum_{m=0}^{M-1}{\left(-1\right)}^{m}\frac{2C_{M-1}^{m}M}{t!}{\left(m+1\right)}^{\frac{t-1}{2}}\left(\frac{\lambda_{\mathrm{BS}}\lambda_{\mathrm{SR}}\rho }{\kappa \Delta }\right)}^{\frac{t+1}{2}}K_{1-t}\left(2\sqrt{\frac{\left(m+1\right)\lambda_{\mathrm{BS}}\lambda_{\mathrm{SR}}\rho }{\kappa \Delta }}\right)\\ & -\sum_{t=0}^{K-1}{\sum_{n=1}^{N}\sum_{m=0}^{M-1}{\left(-1\right)}^{n+m}C_{M-1}^{m}C_{N}^{n}\frac{2M\lambda_{\mathrm{SR}}}{t!}{\left(\frac{\rho }{n\lambda_{\mathrm{SP}}\delta \Delta +\left(m+1\right)\lambda_{\mathrm{SR}}\rho }\right)}^{\frac{1-t}{2}}\left(\frac{\lambda_{\mathrm{BS}}\rho }{\kappa \Delta }\right)}^{\frac{t+1}{2}}\\ & \times K_{1-t}\left(2\sqrt{\frac{\lambda_{\mathrm{BS}}}{\kappa \Delta }\left(n\lambda_{\mathrm{SP}}\delta \Delta +\left(m+1\right)\lambda_{\mathrm{SR}}\rho \right)}\right), \end{array}$$

where $K_{1-t}\left(.\right)$ is modified Bessel function of the second kind ([53], Equation (8.407.1)).

Next, with the same manner as deriving $A_{1}$, we can obtain an exact closed-form expression for $A_{2}$ as

(47) $$\begin{array}{c} A_{2}=1-\sum_{t=0}^{K-1}\frac{2}{t!}{\left(\frac{\lambda_{\mathrm{BR}}\lambda_{\mathrm{RD}}\rho }{\kappa \Delta }\right)}^{\frac{t+1}{2}}K_{1-t}\left(2\sqrt{\frac{\lambda_{\mathrm{BR}}\lambda_{\mathrm{RD}}\rho }{\kappa \Delta }}\right)-\\ \sum_{t=0}^{K-1}\sum_{n=1}^{N}\;{\left(-1\right)}^{n}C_{N}^{n}\frac{2\lambda_{\mathrm{RD}}}{t!}{\left(\frac{\rho }{n\lambda_{\mathrm{RP}}\delta \Delta \;+\;\lambda_{\mathrm{RD}}\rho }\right)}^{\frac{1-t}{2}}\;{\left(\frac{\lambda_{\mathrm{BR}}\rho }{\kappa \Delta }\right)}^{\frac{t+1}{2}}K_{1-t}\;\left(2\sqrt{\frac{\lambda_{\mathrm{BR}}}{\kappa \Delta }\left(\;n\lambda_{\mathrm{RP}}\delta \Delta +\lambda_{\mathrm{RD}}\rho \right)}\right)\;. \end{array}$$

Substituting (46) and (47) into (36), we obtain (34). Next, by replacing $\lambda_{\mathrm{SR}},\;\lambda_{\mathrm{RD}},\;\lambda_{\mathrm{BS}},\;\lambda_{\mathrm{BR}},\lambda_{\mathrm{SP}}$ and $\lambda_{\mathrm{RP}}$ in (34) by $\lambda_{\mathrm{RD}},\;\lambda_{\mathrm{SR}},\;\lambda_{\mathrm{BR}},\;\lambda_{\mathrm{BS}},\lambda_{\mathrm{RP}}$ and $\lambda_{\mathrm{SP}}$, respectively, we can obtain (35). ☐

Next, we provide an exact closed-form expression of the e2e OP for the B-ORS protocol as presented in Lemma 2.

**Lemma** **2.** *When $1-\tau_{D}^{2}\theta >0$, $OP_{B-\mathrm{ORS}}$ can be computed by*

(48) $$\begin{array}{c} \begin{array}{c} OP_{B-\mathrm{ORS}}\\ =1+\sum_{t=0}^{K-1}\sum_{m=1}^{M}{\left(-1\right)}^{m}\frac{2C_{M}^{m}}{t!}{\left(\frac{m\lambda_{\mathrm{BS}}\lambda_{\mathrm{SR}}\rho }{\kappa \Delta }\right)}^{\frac{t+1}{2}}K_{t-1}\left(2\sqrt{\frac{m\lambda_{\mathrm{BS}}\lambda_{\mathrm{SR}}\rho }{\kappa \Delta }}\right)\\ \times {\left[\begin{array}{c} \sum_{t=0}^{K-1}\frac{2}{t!}{\left(\frac{\lambda_{\mathrm{BR}}\lambda_{\mathrm{RD}}\rho }{\kappa \Delta }\right)}^{\frac{t+1}{2}}K_{1-t}\left(2\sqrt{\frac{\lambda_{\mathrm{BR}}\lambda_{\mathrm{RD}}\rho }{\kappa \Delta }}\right)\\ -\sum_{t=0}^{K-1}\sum_{n=1}^{N}{\left(-1\right)}^{n+1}C_{N}^{n}\frac{2\lambda_{\mathrm{RD}}}{t!}{\left(\frac{\rho }{n\lambda_{\mathrm{RP}}\delta \Delta +\lambda_{\mathrm{RD}}\rho }\right)}^{\frac{1-t}{2}}{\left(\frac{\lambda_{\mathrm{BR}}\rho }{\kappa \Delta }\right)}^{\frac{t+1}{2}}K_{1-t}\left(2\sqrt{\frac{\lambda_{\mathrm{BR}}}{\kappa \Delta }\left(n\lambda_{\mathrm{RP}}\delta \Delta +\lambda_{\mathrm{RD}}\rho \right)}\right) \end{array}\right]}^{m}\\ +\sum_{t=0}^{K-1}\sum_{n=1}^{N}\sum_{m=1}^{M}{\left(-1\right)}^{m+n}\frac{2C_{N}^{n}C_{M}^{m}}{t!}{\left(\frac{m\lambda_{\mathrm{BS}}\lambda_{\mathrm{SR}}\rho }{\kappa \Delta }\right)}^{\frac{t+1}{2}}{\left(1+\frac{n\lambda_{\mathrm{SP}}\delta \Delta }{m\lambda_{\mathrm{SR}}\rho }\right)}^{\frac{t-1}{2}}K_{t-1}\left(2\sqrt{\frac{\lambda_{\mathrm{BS}}}{\kappa \Delta }\left(n\lambda_{\mathrm{SP}}\delta \Delta +m\lambda_{\mathrm{SR}}\rho \right)}\right)\\ \times {\left[\begin{array}{c} \sum_{t=0}^{K-1}\frac{2}{t!}{\left(\frac{\lambda_{\mathrm{BR}}\lambda_{\mathrm{RD}}\rho }{\kappa \Delta }\right)}^{\frac{t+1}{2}}K_{1-t}\left(2\sqrt{\frac{\lambda_{\mathrm{BR}}\lambda_{\mathrm{RD}}\rho }{\kappa \Delta }}\right)\;\\ \;\;-\;\sum_{t=0}^{K-1}\sum_{n=1}^{N}{\left(-1\right)}^{n+1}C_{N}^{n}\frac{2\lambda_{\mathrm{RD}}}{t!}{\left(\frac{\rho }{n\lambda_{\mathrm{RP}}\delta \Delta +\lambda_{\mathrm{RD}}\rho }\right)}^{\frac{1-t}{2}}{\left(\frac{\lambda_{\mathrm{BR}}\rho }{\kappa \Delta }\right)}^{\frac{t+1}{2}}K_{1-t}\;\left(2\sqrt{\frac{\lambda_{\mathrm{BR}}}{\kappa \Delta }\left(n\lambda_{\mathrm{RP}}\delta \Delta +\lambda_{\mathrm{RD}}\rho \right)}\right)\; \end{array}\right]}^{m}. \end{array} \end{array}$$

**Proof.**  From (26) and (30), the e2e OP of the B-ORS protocol is expressed by

(49) $$\begin{array}{c} OP_{B-\mathrm{ORS}}=\mathrm{Pr}\left(\min\left(\Psi_{1b},\Psi_{2b}\right)<\theta \right)=\mathrm{Pr}\left(\max_{m=1,2,\ldots ,M}\left(\min\left(\Psi_{1m},\Psi_{2m}\right)\right)<\theta \right). \end{array}$$

We note that the RVs $\Psi_{1m}\left(m=1,2,\ldots ,M\right)$ have a common RV, i.e., $Z_{1}=\min\left(\kappa X_{0}^{\mathrm{sum}},\frac{\delta }{Y_{0}^{\max}}\right).$ Hence, we have to rewrite (49) under the following form:

(50) $$\begin{array}{cc} OP_{B-\mathrm{ORS}} & =\int_{0}^{+\infty }\underset{B_{1}\left(u\right)}{\underset{︸}{\mathrm{Pr}\left(\max_{m=1,2,\ldots ,M}\left(\min\left(\Psi_{1m},\Psi_{2m}\right)\right)<\theta |Z_{1}=u\right)}}f_{Z_{1}}\left(u\right)du\\ & =1+\int_{0}^{+\infty }\frac{\partial B_{1}\left(u\right)}{\partial u}\left(1-F_{Z_{1}}\left(u\right)\right)du. \end{array}$$

Now, we attempt to calculate $B_{1}\left(u\right)$ as marked in (50):

(51) $$\begin{array}{c} B_{1}\left(u\right)={\left[\mathrm{Pr}\left(\min\left(\Psi_{1m},\Psi_{2m}\right)<\theta |Z_{1}=u\right)\right]}^{M}={\left[B_{2}\left(u\right)\right]}^{M}, \end{array}$$

where

(52) $$\begin{array}{cc} B_{2}\left(u\right) & =\mathrm{Pr}\left(\min\left(\Psi_{1m},\Psi_{2m}\right)<\theta |Z_{1}=u\right)=1-\mathrm{Pr}\left(\min\left(\Psi_{1m},\Psi_{2m}\right)\geq \theta |Z_{1}=u\right)\\ & =1-\mathrm{Pr}\left(\gamma_{SR_{m}}\geq \frac{\rho }{\Delta u},\min\left(\kappa X_{m}^{\mathrm{sum}},\frac{\delta }{Y_{m}^{\max}}\right)\gamma_{R_{m}D}\geq \frac{\rho }{\Delta }\right)\\ & =1-\left(1-\mathrm{Pr}\left(\gamma_{SR_{m}}<\frac{\rho }{\Delta u}\right)\right)\left(1-A_{2}\right)\\ & =1-\left(1-A_{2}\right)\exp\left(-\frac{\lambda_{\mathrm{SR}}\rho }{\Delta u}\right). \end{array}$$

In (52), $A_{2}$ is given in (47). Next, substituting (52) into (51), we obtain

(53) $$\begin{array}{c} B_{1}\left(u\right)=1+\sum_{m=1}^{M}{\left(-1\right)}^{m}C_{M}^{m}{\left(1-A_{2}\right)}^{m}\exp\left(-\frac{m\lambda_{\mathrm{SR}}\rho }{\Delta u}\right). \end{array}$$

Differentiating $B_{1}\left(u\right)$ with respect to *u*, yielding

(54) $$\begin{array}{c} \frac{\partial B_{1}\left(u\right)}{\partial u}=\sum_{m=1}^{M}{\left(-1\right)}^{m}C_{M}^{m}\frac{m\lambda_{\mathrm{SR}}\rho }{\Delta }{\left(1-A_{2}\right)}^{m}\frac{1}{u^{2}}\exp\left(-\frac{m\lambda_{\mathrm{SR}}\rho }{\Delta u}\right). \end{array}$$

Substituting (44) and (54) into (50), and then using ([53], Equation (3.471.9)) for the corresponding integrals, we can obtain

(55) $$\begin{array}{cc} OP_{B-\mathrm{ORS}} & =1+\sum_{t=0}^{K-1}\sum_{m=1}^{M}{\left(-1\right)}^{m}\frac{2C_{M}^{m}}{t!}{\left(\frac{m\lambda_{\mathrm{BS}}\lambda_{\mathrm{SR}}\rho }{\kappa \Delta }\right)}^{\frac{t+1}{2}}{\left(1-A_{2}\right)}^{m}K_{t-1}\left(2\sqrt{\frac{m\lambda_{\mathrm{BS}}\lambda_{\mathrm{SR}}\rho }{\kappa \Delta }}\right)\\ & +\sum_{t=0}^{K-1}\sum_{n=1}^{N}\sum_{m=1}^{M}{\left(-1\right)}^{m+n}\frac{2C_{N}^{n}C_{M}^{m}}{t!}{\left(\frac{m\lambda_{\mathrm{BS}}\lambda_{\mathrm{SR}}\rho }{\kappa \Delta }\right)}^{\frac{t+1}{2}}{\left(1-A_{2}\right)}^{m}{\left(1+\frac{n\lambda_{\mathrm{SP}}\delta \Delta }{m\lambda_{\mathrm{SR}}\rho }\right)}^{\frac{t-1}{2}}\\ & \times K_{t-1}\left(2\sqrt{\frac{\lambda_{\mathrm{BS}}}{\kappa \Delta }\left(n\lambda_{\mathrm{SP}}\delta \Delta +m\lambda_{\mathrm{SR}}\rho \right)}\right). \end{array}$$

Next, substituting (47) into (55), we obtain (48).

The derivation of (48) is different from that of (34) and (35) due to the dependence of the end-to-end SNRs. ☐

**Lemma** **3.** *When $1-\tau_{D}^{2}\theta >0$, $OP_{C-\mathrm{ORS}}$ can be expressed by an exact expression as in* (56)

(56) $$\begin{array}{c} OP_{C-\mathrm{ORS}}=1\\ -\sum_{m=0}^{M-1}{\sum_{t=0}^{K-1}\sum_{n=0}^{N}\sum_{w=0}^{K-1}\sum_{q=0}^{N}{\left(-1\right)}^{m+n+q}C_{M-1}^{m}\frac{C_{N}^{n}C_{N}^{q}}{t!w!}\left(\frac{\lambda_{\mathrm{BS}}}{\kappa }\right)}^{t}{\left(\frac{\lambda_{\mathrm{BR}}}{\kappa }\right)}^{w}\frac{M\lambda_{\mathrm{RD}}}{\lambda_{\mathrm{RD}}+m\Omega }\\ \times \int_{0}^{+\infty }\int_{0}^{z_{2}}\left(\frac{\lambda_{\mathrm{BS}}}{\kappa }z_{1}^{t}-n\lambda_{\mathrm{SP}}\delta z_{1}^{t-2}-tz_{1}^{t-1}\right)\left(\frac{\lambda_{\mathrm{BR}}}{\kappa }z_{2}^{w}-q\lambda_{\mathrm{RP}}\delta z_{2}^{w-2}-wz_{2}^{w-1}\right)\\ \times \exp\left(-\frac{\lambda_{\mathrm{SR}}\rho }{\Delta }\frac{1}{z_{1}}\right)\exp\left(-\frac{\left(\lambda_{\mathrm{RD}}+m\Omega \right)\rho }{\Delta }\frac{1}{z_{2}}\right)\exp\left(-\frac{\lambda_{\mathrm{BS}}}{\kappa }z_{1}-\frac{n\lambda_{\mathrm{SP}}\delta }{z_{1}}\right)\\ \times \exp\left(-\frac{\lambda_{\mathrm{BR}}}{\kappa }z_{2}-\frac{q\lambda_{\mathrm{RP}}\delta }{z_{2}}\right)dz_{1}dz_{2}\\ -\sum_{m=0}^{M-1}{\sum_{t=0}^{K-1}\sum_{n=0}^{N}\sum_{w=0}^{K-1}\sum_{q=0}^{N}{\left(-1\right)}^{m+n+q}C_{M-1}^{m}\frac{C_{N}^{n}C_{N}^{q}}{t!w!}\left(\frac{\lambda_{\mathrm{BS}}}{\kappa }\right)}^{t}{\left(\frac{\lambda_{\mathrm{BR}}}{\kappa }\right)}^{w}\frac{m}{m+1}\frac{M\lambda_{\mathrm{SR}}}{\lambda_{\mathrm{RD}}+m\Omega }\\ \times \int_{0}^{+\infty }\int_{0}^{z_{2}}\left(\frac{\lambda_{\mathrm{BS}}}{\kappa }z_{1}^{t}-n\lambda_{\mathrm{SP}}\delta z_{1}^{t-2}-tz_{1}^{t-1}\right)\left(\frac{\lambda_{\mathrm{BR}}}{\kappa }z_{2}^{w}-q\lambda_{\mathrm{RP}}\delta z_{2}^{w-2}-wz_{2}^{w-1}\right)\\ \times \exp\left(-\frac{\left(m+1\right)\Omega \rho }{\Delta }\frac{1}{z_{1}}\right)\exp\left(-\frac{\lambda_{\mathrm{BS}}}{\kappa }z_{1}-\frac{n\lambda_{\mathrm{SP}}\delta }{z_{1}}\right)\exp\left(-\frac{\lambda_{\mathrm{BS}}}{\kappa }z_{2}-\frac{q\lambda_{\mathrm{RP}}\delta }{z_{2}}\right)dz_{1}dz_{2}\\ -\sum_{m=0}^{M-1}{\sum_{t=0}^{K-1}\sum_{n=0}^{N}\sum_{w=0}^{K-1}\sum_{q=0}^{N}{\left(-1\right)}^{m+n+q}C_{M-1}^{m}\frac{C_{N}^{n}C_{N}^{q}}{t!w!}\left(\frac{\lambda_{\mathrm{BS}}}{\kappa }\right)}^{t}{\left(\frac{\lambda_{\mathrm{BR}}}{\kappa }\right)}^{w}\frac{M\lambda_{\mathrm{SR}}}{\lambda_{\mathrm{SR}}+m\Omega }\\ \times \int_{0}^{+\infty }\int_{0}^{z_{1}}\left(\frac{\lambda_{\mathrm{BS}}}{\kappa }z_{1}^{t}-n\lambda_{\mathrm{SP}}\delta z_{1}^{t-2}-tz_{1}^{t-1}\right)\left(\frac{\lambda_{\mathrm{BR}}}{\kappa }z_{2}^{w}-q\lambda_{\mathrm{RP}}\delta z_{2}^{w-2}-wz_{2}^{w-1}\right)\\ \times \exp\left(-\frac{\lambda_{\mathrm{RD}}\rho }{\Delta }\frac{1}{z_{2}}\right)\exp\left(-\frac{\left(\lambda_{\mathrm{SR}}+m\Omega \right)\rho }{\Delta }\frac{1}{z_{1}}\right)\exp\left(-\frac{\lambda_{\mathrm{BS}}}{\kappa }z_{1}-\frac{n\lambda_{\mathrm{SP}}\delta }{z_{1}}\right)\\ \times \exp\left(-\frac{\lambda_{\mathrm{BR}}}{\kappa }z_{2}-\frac{q\lambda_{\mathrm{RP}}\delta }{z_{2}}\right)dz_{1}dz_{2}\\ -\sum_{m=0}^{M-1}{\sum_{t=0}^{K-1}\sum_{n=0}^{N}\sum_{w=0}^{K-1}\sum_{q=0}^{N}{\left(-1\right)}^{m+n+q}C_{M-1}^{m}\frac{C_{N}^{n}C_{N}^{q}}{t!w!}\left(\frac{\lambda_{\mathrm{BS}}}{\kappa }\right)}^{t}{\left(\frac{\lambda_{\mathrm{BR}}}{\kappa }\right)}^{w}\frac{m}{m+1}\frac{M\lambda_{\mathrm{RD}}}{\lambda_{\mathrm{SR}}+m\Omega }\\ \times \int_{0}^{+\infty }\int_{0}^{z_{2}}\left(\frac{\lambda_{\mathrm{BS}}}{\kappa }z_{1}^{t}-n\lambda_{\mathrm{SP}}\delta z_{1}^{t-2}-tz_{1}^{t-1}\right)\left(\frac{\lambda_{\mathrm{BR}}}{\kappa }z_{2}^{w}-q\lambda_{\mathrm{RP}}\delta z_{2}^{w-2}-wz_{2}^{w-1}\right)\\ \times \exp\left(-\frac{\left(m+1\right)\Omega \rho }{\Delta }\frac{1}{z_{2}}\right)\exp\left(-\frac{\lambda_{\mathrm{BS}}}{\kappa }z_{1}-\frac{n\lambda_{\mathrm{SP}}\delta }{z_{1}}\right)\exp\left(-\frac{\lambda_{\mathrm{BS}}}{\kappa }z_{2}-\frac{q\lambda_{\mathrm{RP}}\delta }{z_{2}}\right)dz_{1}dz_{2}. \end{array}$$

**Proof.**  In the C-ORS protocol, the end-to-end OP can be calculated by

(57) $$\begin{array}{cc} OP_{C-\mathrm{ORS}} & =\mathrm{Pr}\left(\min\left(\min\left(\kappa X_{0}^{\mathrm{sum}},\frac{\delta }{Y_{0}^{\max}}\right)\gamma_{SR_{c}},\min\left(\kappa X_{c}^{\mathrm{sum}},\frac{\delta }{Y_{c}^{\max}}\right)\gamma_{R_{c}D}\right)<\frac{\rho }{\Delta }\right)\\ & =\mathrm{Pr}\left(\min\left(Z_{1}\gamma_{SR_{c}},Z_{2}\gamma_{R_{c}D}\right)<\frac{\rho }{\Delta }\right)\\ & =1-\mathrm{Pr}\left(\gamma_{SR_{c}}\geq \frac{\rho }{\Delta Z_{1}},\gamma_{R_{c}D}\geq \frac{\rho }{\Delta Z_{2}}\right), \end{array}$$

where $Z_{2}=\min\left(\kappa X_{c}^{\mathrm{sum}},\frac{\delta }{Y_{c}^{\max}}\right),$ and the CDF of $Z_{2}$ is given, similar to $Z_{1}$ in (44):

(58) $$\begin{array}{cc} F_{Z_{2}}\left(x\right) & =1-\sum_{t=0}^{K-1}{\frac{1}{t!}\left(\frac{\lambda_{\mathrm{BR}}}{\kappa }\right)}^{t}x^{t}\exp\left(-\frac{\lambda_{\mathrm{BR}}}{\kappa }x\right)\\ & -\sum_{t=0}^{K-1}{\sum_{n=1}^{N}{\left(-1\right)}^{n}\frac{C_{N}^{n}}{t!}\left(\frac{\lambda_{\mathrm{BR}}}{\kappa }\right)}^{t}x^{t}\exp\left(-\frac{\lambda_{\mathrm{BR}}}{\kappa }x-\frac{n\lambda_{\mathrm{RP}}\delta }{x}\right). \end{array}$$

Since $\gamma_{SR_{c}}$ and $\gamma_{R_{c}D}$ are not independent, the method in [49] can be used to calculate $OP_{C-\mathrm{ORS}}$. At first, using ([49], Equation (D.2)), we have

(59) $$\begin{array}{c} \mathrm{Pr}\left(\gamma_{SR_{c}}\geq u_{1},\gamma_{R_{c}D}\geq u_{2}\right)=\int_{0}^{+\infty }\frac{\partial G\left(z\right)}{\partial z}\frac{f_{T_{\max}}\left(z\right)}{f_{T_{m}}\left(z\right)}dz, \end{array}$$

where $T_{m}=\min\left(\gamma_{SR_{m}},\gamma_{R_{m}D}\right),$ $T_{\max}=\max_{m=1,2,\ldots ,M}\left(T_{m}\right),$ and $G\left(z\right)=\mathrm{Pr}(\gamma_{SR_{m}}\geq u_{1},\gamma_{R_{m}D}\geq u_{2}$, $\min\left(\gamma_{SR_{m}},\gamma_{R_{m}D}\right)<z).$

Because the CDF of $T_{m}$ is $F_{T_{\max}}\left(z\right)=1-\exp\left(-\left(\lambda_{\mathrm{SR}}+\lambda_{\mathrm{RD}}\right)x\right),$ its PDF is obtained as

(60) $$\begin{array}{c} f_{T_{\max}}\left(z\right)=\left(\lambda_{\mathrm{SR}}+\lambda_{\mathrm{RD}}\right)\exp\left(-\left(\lambda_{\mathrm{SR}}+\lambda_{\mathrm{RD}}\right)z\right)=\Omega \exp\left(-\Omega z\right), \end{array}$$

where $\Omega =\lambda_{\mathrm{SR}}+\lambda_{\mathrm{RD}}.$

Then, the PDF of $T_{max}$ can obtained, similar to (40), as

(61) $$\begin{array}{c} f_{T_{\max}}\left(z\right)=\sum_{m=0}^{M-1}{\left(-1\right)}^{m}C_{M-1}^{m}M\Omega \exp\left(-\left(m+1\right)\Omega z\right). \end{array}$$

Considering the probability $G\left(z\right)$ in (59); using ([49], Equation (D7)–(D8)), we have

- **Case 1:**$u_{1}\geq u_{2}$
  (62) $$\begin{array}{c} \frac{\partial G\left(z\right)}{\partial z}=\left\{\begin{array}{cc} 0, & \mathrm{if}\;z<u_{2},\\ \lambda_{\mathrm{RD}}\exp\left(-\lambda_{\mathrm{SR}}u_{1}\right)\exp\left(-\lambda_{\mathrm{RD}}z\right), & \mathrm{if}\;u_{2}\leq z<u_{1},\\ \Omega \exp\left(-\Omega z\right), & \mathrm{if}\;u_{1}\leq z. \end{array}\right. \end{array}$$
  Plugging (59)–(62) together, and after some manipulations, the following can be obtained:
  (63) $$\begin{array}{c} \mathrm{Pr}\left(\gamma_{SR_{c}}\geq u_{1},\gamma_{R_{c}D}\geq u_{2}|u_{1}\geq u_{2}\right)\\ =\sum_{m=0}^{M-1}{\left(-1\right)}^{m}C_{M-1}^{m}M\left[\begin{array}{c} \int_{u_{2}}^{u_{1}}\lambda_{\mathrm{RD}}\exp\left(-\lambda_{\mathrm{SR}}u_{1}\right)\exp\left(-\lambda_{\mathrm{RD}}z\right)\exp\left(-m\Omega z\right)dz\\ +\int_{u_{1}}^{+\infty }\Omega \exp\left(-\left(m+1\right)\Omega z\right)dz \end{array}\right]\\ =\sum_{m=0}^{M-1}{\left(-1\right)}^{m}C_{M-1}^{m}M\left[\begin{array}{c} \frac{\lambda_{\mathrm{RD}}}{\lambda_{\mathrm{RD}}+m\Omega }\exp\left(-\lambda_{\mathrm{SR}}u_{1}\right)\exp\left(-\left(\lambda_{\mathrm{RD}}+m\Omega \right)u_{2}\right)\\ +\frac{m}{m+1}\frac{\lambda_{\mathrm{SR}}}{\lambda_{\mathrm{RD}}+m\Omega }\exp\left(-\left(m+1\right)\Omega u_{1}\right) \end{array}\right]. \end{array}$$
- **Case 2:**$u_{1}<u_{2}$
  (64) $$\begin{array}{c} \frac{\partial G\left(z\right)}{\partial z}=\left\{\begin{array}{cc} 0, & \mathrm{if}\;z<u_{1},\\ \lambda_{\mathrm{SR}}\exp\left(-\lambda_{\mathrm{RD}}u_{2}\right)\exp\left(-\lambda_{\mathrm{SR}}z\right), & \mathrm{if}\;u_{1}\leq z<u_{2},\\ \Omega \exp\left(-\Omega z\right), & \mathrm{if}\;u_{2}\leq z. \end{array}\right. \end{array}$$
  Similarly, we obtain
  (65) $$\begin{array}{c} \mathrm{Pr}\left(\gamma_{SR_{c}}\geq u_{1},\gamma_{R_{c}D}\geq u_{2}|u_{2}>u_{1}\right)\\ =\sum_{m=0}^{M-1}{\left(-1\right)}^{m}C_{M-1}^{m}M\left[\begin{array}{c} \frac{\lambda_{\mathrm{SR}}}{\lambda_{\mathrm{SR}}+m\Omega }\exp\left(-\lambda_{\mathrm{RD}}u_{2}\right)\exp\left(-\left(\lambda_{\mathrm{SR}}+m\Omega \right)u_{1}\right)\\ +\frac{m}{m+1}\frac{\lambda_{\mathrm{RD}}}{\lambda_{\mathrm{SR}}+m\Omega }\exp\left(-\left(m+1\right)\Omega u_{2}\right) \end{array}\right]. \end{array}$$
  Now, with $u_{1}=\frac{\rho }{\Delta Z_{1}}$ and $u_{2}=\frac{\rho }{\Delta Z_{2}}$, the following are respectively obtained:
  (66) $$\begin{array}{c} \mathrm{Pr}\left(\gamma_{SR_{c}}\geq \frac{\rho }{\Delta z_{1}},\gamma_{R_{c}D}\geq \frac{\rho }{\Delta z_{2}}|z_{2}\geq z_{1}\right)\\ =\sum_{m=0}^{M-1}{\left(-1\right)}^{m}C_{M-1}^{m}M\times \left[\begin{array}{c} \frac{\lambda_{\mathrm{RD}}}{\lambda_{\mathrm{RD}}+m\Omega }\exp\left(-\frac{\lambda_{\mathrm{SR}}\rho }{\Delta }\frac{1}{z_{1}}\right)\exp\left(-\frac{\left(\lambda_{\mathrm{RD}}+m\Omega \right)\rho }{\Delta }\frac{1}{z_{2}}\right)\\ +\frac{m}{m+1}\frac{\lambda_{\mathrm{SR}}}{\lambda_{\mathrm{RD}}+m\Omega }\exp\left(-\frac{\left(m+1\right)\Omega \rho }{\Delta }\frac{1}{z_{1}}\right) \end{array}\right], \end{array}$$
  (67) $$\begin{array}{c} \mathrm{Pr}\left(\gamma_{SR_{c}}\geq \frac{\rho }{\Delta z_{1}},\gamma_{R_{c}D}\geq \frac{\rho }{\Delta z_{2}}|z_{1}>z_{2}\right)\\ =\sum_{m=0}^{M-1}{\left(-1\right)}^{m}C_{M-1}^{m}M\left[\begin{array}{c} \frac{\lambda_{\mathrm{SR}}}{\lambda_{\mathrm{SR}}+m\Omega }\exp\left(-\frac{\lambda_{\mathrm{RD}}\rho }{\Delta }\frac{1}{z_{2}}\right)\exp\left(-\frac{\left(\lambda_{\mathrm{SR}}+m\Omega \right)\rho }{\Delta }\frac{1}{z_{1}}\right)\\ +\frac{m}{m+1}\frac{\lambda_{\mathrm{RD}}}{\lambda_{\mathrm{SR}}+m\Omega }\exp\left(-\frac{\left(m+1\right)\Omega \rho }{\Delta }\frac{1}{z_{2}}\right) \end{array}\right]. \end{array}$$

Moreover, $OP_{C-\mathrm{ORS}}$ in (57) can be formulated as

(68) $$\begin{array}{cc} OP_{C-\mathrm{ORS}} & =1-\int_{0}^{+\infty }\int_{0}^{z_{2}}\mathrm{Pr}\left(\gamma_{SR_{c}}\geq \frac{\rho }{\Delta z_{1}},\gamma_{R_{c}D}\geq \frac{\rho }{\Delta z_{2}}|z_{2}\geq z_{1}\right)f_{Z_{1}}\left(z_{1}\right)f_{Z_{2}}\left(z_{2}\right)dz_{1}dz_{2}\\ & -\int_{0}^{+\infty }\int_{0}^{z_{1}}\mathrm{Pr}\left(\gamma_{SR_{c}}\geq \frac{\rho }{\Delta z_{1}},\gamma_{R_{c}D}\geq \frac{\rho }{\Delta z_{2}}|z_{1}\geq z_{2}\right)f_{Z_{1}}\left(z_{1}\right)f_{Z_{2}}\left(z_{2}\right)dz_{1}dz_{2}, \end{array}$$

where the PDFs of $Z_{1}$ and $Z_{2}$ can be obtained from their CDFs, i.e.,

(69) $$\begin{array}{c} f_{Z_{1}}\left(z_{1}\right)\;=\;\sum_{t=0}^{K-1}{\sum_{n=0}^{N}{\left(-1\right)}^{n}\frac{C_{N}^{n}}{t!}\left(\frac{\lambda_{\mathrm{BS}}}{\kappa }\right)}^{t}\left(\frac{\lambda_{\mathrm{BS}}}{\kappa }z_{1}^{t}-n\lambda_{\mathrm{SP}}\delta z_{1}^{t-2}-tz_{1}^{t-1}\right)\exp\;\left(\;-\frac{\lambda_{\mathrm{BS}}}{\kappa }z_{1}-\frac{n\lambda_{\mathrm{SP}}\delta }{z_{1}}\right), \end{array}$$

(70) $$\begin{array}{c} f_{Z_{2}}\left(z_{2}\right)\;=\;\sum_{w=0}^{K-1}{\sum_{q=0}^{N}{\left(-1\right)}^{q}\;\frac{C_{N}^{q}}{w!}\;\left(\;\frac{\lambda_{\mathrm{BR}}}{\kappa }\;\right)}^{w}\;\left(\;\frac{\lambda_{\mathrm{BR}}}{\kappa }z_{2}^{w}-q\lambda_{\mathrm{RP}}\delta z_{2}^{w-2}-wz_{2}^{w-1}\;\right)\;\exp\;\left(\;-\frac{\lambda_{\mathrm{BR}}}{\kappa }z_{2}-\frac{q\lambda_{\mathrm{RP}}\delta }{z_{2}}\right)\;. \end{array}$$

Substituting (66), (67), (69), and (70) into (68), Equation (56) can be obtained to finish the proof.

However, the exact expression of $OP_{C-\mathrm{ORS}}$ is still in integral form, which is difficult to use for designing and optimizing the system. This motivates us to derive the approximate closed-form expression for $OP_{C-\mathrm{ORS}}$. ☐

**Lemma** **4.** *When $1-\tau_{D}^{2}\theta >0$, $OP_{C-\mathrm{ORS}}$ can be approximated by a closed-form expression as given in (71) at the top of next page.*

(71) $$\begin{array}{c} \begin{array}{c} OP_{C-\mathrm{ORS}}\;\approx 1-\left\{\begin{array}{c} \sum_{t=0}^{K-1}\sum_{m=1}^{M}{\left(-1\right)}^{m-1}\frac{C_{M}^{m}}{t!}{\left(\frac{\lambda_{\mathrm{SR}}\lambda_{\mathrm{BS}}\rho }{\kappa \Delta }\right)}^{\frac{t+1}{2}}\frac{2m\lambda_{\mathrm{RD}}}{m\left(\lambda_{\mathrm{SR}}+\lambda_{\mathrm{RD}}\right)-\lambda_{\mathrm{SR}}}K_{1-t}\left(2\sqrt{\frac{\lambda_{\mathrm{SR}}\lambda_{\mathrm{BS}}\rho }{\kappa \Delta }}\right)\\ +\sum_{t=0}^{K-1}\sum_{m=1}^{M}{\left(-1\right)}^{m-1}\frac{C_{M}^{m}}{t!}{\left(\frac{m\left(\lambda_{\mathrm{SR}}+\lambda_{\mathrm{RD}}\right)\lambda_{\mathrm{BS}}\rho }{\kappa \Delta }\right)}^{\frac{t+1}{2}}\\ \times \frac{2\left(m-1\right)\lambda_{\mathrm{SR}}}{m\left(\lambda_{\mathrm{SR}}+\lambda_{\mathrm{RD}}\right)-\lambda_{\mathrm{SR}}}K_{1-t}\left(2\sqrt{\frac{m\left(\lambda_{\mathrm{SR}}+\lambda_{\mathrm{RD}}\right)\lambda_{\mathrm{BS}}\rho }{\kappa \Delta }}\right)\\ +\sum_{t=0}^{K-1}\sum_{n=1}^{N}\sum_{m=1}^{M}{\left(-1\right)}^{m+n-1}\frac{C_{N}^{n}C_{M}^{m}}{t!}{\left(\frac{\lambda_{\mathrm{BS}}\rho }{\kappa \Delta }\right)}^{\frac{t+1}{2}}\;{\left(\frac{\rho }{n\lambda_{\mathrm{SP}}\delta \Delta +\lambda_{\mathrm{SR}}\rho }\right)}^{\frac{1-t}{2}}\\ \times \frac{2m\lambda_{\mathrm{SR}}\lambda_{\mathrm{RD}}}{m\left(\lambda_{\mathrm{SR}}+\lambda_{\mathrm{RD}}\right)-\lambda_{\mathrm{SR}}}K_{1-t}\left(2\sqrt{\frac{\lambda_{\mathrm{BS}}}{\kappa \Delta }\left(n\lambda_{\mathrm{SP}}\delta \Delta +\lambda_{\mathrm{SR}}\rho \right)}\right)\\ +\sum_{t=0}^{K-1}\sum_{n=1}^{N}\sum_{m=1}^{M}{\left(-1\right)}^{m+n-1}\frac{C_{N}^{n}C_{M}^{m}}{t!}{\left(\frac{\lambda_{\mathrm{BS}}\rho }{\kappa \Delta }\right)}^{\frac{t+1}{2}}\;{\left(\frac{\rho }{n\lambda_{\mathrm{SP}}\delta \Delta +m\left(\lambda_{\mathrm{SR}}+\lambda_{\mathrm{RD}}\right)\rho }\right)}^{\frac{1-t}{2}}\\ \times \frac{2m\left(m-1\right)\lambda_{\mathrm{SR}}\left(\lambda_{\mathrm{SR}}+\lambda_{\mathrm{RD}}\right)}{m\left(\lambda_{\mathrm{SR}}+\lambda_{\mathrm{RD}}\right)-\lambda_{\mathrm{SR}}}K_{1-t}\left(2\sqrt{\frac{\lambda_{\mathrm{BS}}}{\kappa \Delta }\left(n\lambda_{\mathrm{SP}}\delta \Delta +m\left(\lambda_{\mathrm{SR}}+\lambda_{\mathrm{RD}}\right)\rho \right)}\right) \end{array}\right\} \end{array} \end{array}$$

$$\begin{array}{c} \;\;\;\times \left\{\begin{array}{c} \sum_{t=0}^{K-1}\sum_{m=1}^{M}{\left(-1\right)}^{m-1}\frac{C_{M}^{m}}{t!}{\left(\frac{\lambda_{\mathrm{RD}}\lambda_{\mathrm{BR}}\rho }{\kappa \Delta }\right)}^{\frac{t+1}{2}}\frac{2m\lambda_{\mathrm{SR}}}{m\left(\lambda_{\mathrm{SR}}+\lambda_{\mathrm{RD}}\right)-\lambda_{\mathrm{RD}}}K_{1-t}\left(2\sqrt{\frac{\lambda_{\mathrm{RD}}\lambda_{\mathrm{BR}}\rho }{\kappa \Delta }}\right)\\ +\sum_{t=0}^{K-1}\sum_{m=1}^{M}{\left(-1\right)}^{m-1}\frac{C_{M}^{m}}{t!}{\left(\frac{m\left(\lambda_{\mathrm{SR}}+\lambda_{\mathrm{RD}}\right)\lambda_{\mathrm{BR}}\rho }{\kappa \Delta }\right)}^{\frac{t+1}{2}}\\ \times \frac{2\left(m-1\right)\lambda_{\mathrm{RD}}}{m\left(\lambda_{\mathrm{SR}}+\lambda_{\mathrm{RD}}\right)-\lambda_{\mathrm{RD}}}K_{1-t}\left(2\sqrt{\frac{m\left(\lambda_{\mathrm{SR}}+\lambda_{\mathrm{RD}}\right)\lambda_{\mathrm{BR}}\rho }{\kappa \Delta }}\right)\\ +\sum_{t=0}^{K-1}\sum_{n=1}^{N}\sum_{m=1}^{M}{\left(-1\right)}^{m+n-1}\frac{C_{N}^{n}C_{M}^{m}}{t!}{\left(\frac{\lambda_{\mathrm{BR}}\rho }{\kappa \Delta }\right)}^{\frac{t+1}{2}}\;{\left(\frac{\rho }{n\lambda_{\mathrm{RP}}\delta \Delta +\lambda_{\mathrm{RD}}\rho }\right)}^{\frac{1-t}{2}}\\ \times \frac{2m\lambda_{\mathrm{SR}}\lambda_{\mathrm{RD}}}{m\left(\lambda_{\mathrm{SR}}+\lambda_{\mathrm{RD}}\right)-\lambda_{\mathrm{RD}}}K_{1-t}\left(2\sqrt{\frac{\lambda_{\mathrm{BR}}}{\kappa \Delta }\left(n\lambda_{\mathrm{RP}}\delta \Delta +\lambda_{\mathrm{RD}}\rho \right)}\right)\\ +\sum_{t=0}^{K-1}\sum_{n=1}^{N}\sum_{m=1}^{M}{\left(-1\right)}^{m+n-1}\frac{C_{N}^{n}C_{M}^{m}}{t!}{\left(\frac{\lambda_{\mathrm{BR}}\rho }{\kappa \Delta }\right)}^{\frac{t+1}{2}}\;{\left(\frac{\rho }{n\lambda_{\mathrm{RP}}\delta \Delta +m\left(\lambda_{\mathrm{SR}}+\lambda_{\mathrm{RD}}\right)}\right)}^{\frac{1-t}{2}}\\ \times \frac{2m\left(m-1\right)\lambda_{\mathrm{SR}}\left(\lambda_{\mathrm{SR}}+\lambda_{\mathrm{RD}}\right)}{m\left(\lambda_{\mathrm{SR}}+\lambda_{\mathrm{RD}}\right)-\lambda_{\mathrm{RD}}}K_{1-t}\left(2\sqrt{\frac{\lambda_{\mathrm{BS}}}{\kappa \Delta }\left(n\lambda_{\mathrm{RP}}\delta \Delta +m\left(\lambda_{\mathrm{SR}}+\lambda_{\mathrm{RD}}\right)\rho \right)}\right) \end{array}\right\}. \end{array}$$

**Proof.**  Firstly, relaxing the dependence between $\gamma_{SR_{c}}$ and $\gamma_{R_{c}D}$, we have the following approximation:

(72) $$\begin{array}{cc} OP_{C-\mathrm{ORS}} & \approx 1-\mathrm{Pr}\left(\gamma_{SR_{c}}\geq \frac{\rho }{\Delta Z_{1}}\right)\mathrm{Pr}\left(\gamma_{R_{c}D}\geq \frac{\rho }{\Delta Z_{2}}\right)\\ & \approx 1-\mathrm{Pr}\left(Z_{1}\geq \frac{\rho }{\Delta \gamma_{SR_{c}}}\right)\mathrm{Pr}\left(Z_{2}\geq \frac{\rho }{\Delta \gamma_{R_{c}D}}\right). \end{array}$$

Our next objective is to calculate $\mathrm{Pr}\left(Z_{1}\geq \frac{\rho }{\Delta \gamma_{SR_{c}}}\right)$ and $\mathrm{Pr}\left(Z_{2}\geq \frac{\rho }{\Delta \gamma_{R_{c}D}}\right)$, i.e.,

(73) $$\begin{array}{cc} \mathrm{Pr}\left(Z_{1}\geq \frac{\rho }{\Delta \gamma_{SR_{c}}}\right) & =\int_{0}^{+\infty }\left(1-F_{Z_{1}}\left(\frac{\rho }{\Delta y}\right)\right)f_{\gamma_{SR_{c}}}\left(y\right)dy,\\ \mathrm{Pr}\left(Z_{2}\geq \frac{\rho }{\Delta \gamma_{R_{c}D}}\right) & =\int_{0}^{+\infty }\left(1-F_{Z_{2}}\left(\frac{\rho }{\Delta y}\right)\right)f_{\gamma_{R_{c}D}}\left(y\right)dy. \end{array}$$

Using ([34], Equation (2)), we obtain PDF of $\gamma_{SR_{c}}$ and $\gamma_{R_{c}D}$, respectively, as

(74) $$\begin{array}{cc} f_{\gamma_{SR_{c}}}\left(y\right) & =\sum_{m=1}^{M}{\left(-1\right)}^{m-1}C_{M}^{m}\;\frac{m\lambda_{\mathrm{SR}}\lambda_{\mathrm{RD}}}{m\left(\lambda_{\mathrm{SR}}+\lambda_{\mathrm{RD}}\right)-\lambda_{\mathrm{SR}}}\exp\left(-\lambda_{\mathrm{SR}}y\right)\\ & +\sum_{m=1}^{M}{\left(-1\right)}^{m-1}C_{M}^{m}\;\frac{m\left(m-1\right)\lambda_{\mathrm{SR}}\left(\lambda_{\mathrm{SR}}+\lambda_{\mathrm{RD}}\right)}{m\left(\lambda_{\mathrm{SR}}+\lambda_{\mathrm{RD}}\right)-\lambda_{\mathrm{SR}}}\exp\left(-m\left(\lambda_{\mathrm{SR}}+\lambda_{\mathrm{RD}}\right)y\right),\\ f_{\gamma_{R_{c}D}}\left(y\right) & =\sum_{m=1}^{M}{\left(-1\right)}^{m-1}C_{M}^{m}\;\frac{m\lambda_{\mathrm{SR}}\lambda_{\mathrm{RD}}}{m\left(\lambda_{\mathrm{SR}}+\lambda_{\mathrm{RD}}\right)-\lambda_{\mathrm{RD}}}\exp\left(-\lambda_{\mathrm{RD}}y\right)\\ & +\sum_{m=1}^{M}{\left(-1\right)}^{m-1}C_{M}^{m}\;\frac{m\left(m-1\right)\lambda_{\mathrm{SR}}\left(\lambda_{\mathrm{SR}}+\lambda_{\mathrm{RD}}\right)}{m\left(\lambda_{\mathrm{SR}}+\lambda_{\mathrm{RD}}\right)-\lambda_{\mathrm{RD}}}\exp\left(-m\left(\lambda_{\mathrm{SR}}+\lambda_{\mathrm{RD}}\right)y\right). \end{array}$$

Combining (44) and (74), which yields

(75) $$\begin{array}{cc} \mathrm{Pr}\left(Z_{1}\geq \frac{\rho }{\Delta \gamma_{SR_{c}}}\right) & =\sum_{t=0}^{K-1}\sum_{m=1}^{M}{\left(-1\right)}^{m-1}\frac{C_{M}^{m}}{t!}{\left(\frac{\lambda_{\mathrm{BS}}\rho }{\kappa \Delta }\right)}^{t}\frac{m\lambda_{\mathrm{SR}}\lambda_{\mathrm{RD}}}{m\left(\lambda_{\mathrm{SR}}+\lambda_{\mathrm{RD}}\right)-\lambda_{\mathrm{SR}}}\\ & \times \int_{0}^{+\infty }\frac{1}{y^{t}}\exp\left(-\frac{\lambda_{\mathrm{BS}}\rho }{\kappa \Delta }\frac{1}{y}\right)\exp\left(-\lambda_{\mathrm{SR}}y\right)dy\\ & +\sum_{t=0}^{K-1}\sum_{m=1}^{M}{\left(-1\right)}^{m-1}\frac{C_{M}^{m}}{t!}{\left(\frac{\lambda_{\mathrm{BS}}\rho }{\kappa \Delta }\right)}^{t}\frac{m\left(m-1\right)\lambda_{\mathrm{SR}}\left(\lambda_{\mathrm{SR}}+\lambda_{\mathrm{RD}}\right)}{m\left(\lambda_{\mathrm{SR}}+\lambda_{\mathrm{RD}}\right)-\lambda_{\mathrm{SR}}}\\ & \times \int_{0}^{+\infty }\frac{1}{y^{t}}\exp\left(-\frac{\lambda_{\mathrm{BS}}\rho }{\kappa \Delta }\frac{1}{y}\right)\exp\left(-m\left(\lambda_{\mathrm{SR}}+\lambda_{\mathrm{RD}}\right)y\right)dy\\ & +\sum_{t=0}^{K-1}\sum_{n=1}^{N}\sum_{m=1}^{M}{\left(-1\right)}^{m+n-1}\frac{C_{N}^{n}C_{M}^{m}}{t!}{\left(\frac{\lambda_{\mathrm{BS}}\rho }{\kappa \Delta }\right)}^{t}\;\frac{m\lambda_{\mathrm{SR}}\lambda_{\mathrm{RD}}}{m\left(\lambda_{\mathrm{SR}}+\lambda_{\mathrm{RD}}\right)-\lambda_{\mathrm{SR}}}\\ & \times \int_{0}^{+\infty }\frac{1}{y^{t}}\exp\left(-\frac{\lambda_{\mathrm{BS}}\rho }{\kappa \Delta }\frac{1}{y}\right)\exp\left(-\left(\frac{n\lambda_{\mathrm{SP}}\delta \Delta }{\rho }+\lambda_{\mathrm{SR}}\right)y\right)dy\\ & +\sum_{t=0}^{K-1}\sum_{n=1}^{N}\sum_{m=1}^{M}{\left(-1\right)}^{m+n-1}\frac{C_{N}^{n}C_{M}^{m}}{t!}{\left(\frac{\lambda_{\mathrm{BS}}\rho }{\kappa \Delta }\right)}^{t}\;\frac{m\left(m-1\right)\lambda_{\mathrm{SR}}\left(\lambda_{\mathrm{SR}}+\lambda_{\mathrm{RD}}\right)}{m\left(\lambda_{\mathrm{SR}}+\lambda_{\mathrm{RD}}\right)-\lambda_{\mathrm{SR}}}\\ & \times \int_{0}^{+\infty }\frac{1}{y^{t}}\exp\left(-\frac{\lambda_{\mathrm{BS}}\rho }{\kappa \Delta }\frac{1}{y}\right)\exp\left(-\left(\frac{n\lambda_{\mathrm{SP}}\delta \Delta }{\rho }+m\left(\lambda_{\mathrm{SR}}+\lambda_{\mathrm{RD}}\right)\right)y\right). \end{array}$$

Using ([53], Equation (3.471.9)) for the corresponding integrals, and, after some manipulations, we obtain

(76) $$\begin{array}{cc} \mathrm{Pr}\left(Z_{1}\geq \frac{\rho }{\Delta \gamma_{SR_{c}}}\right) & =\sum_{t=0}^{K-1}\sum_{m=1}^{M}{\left(-1\right)}^{m-1}\frac{C_{M}^{m}}{t!}{\left(\frac{\lambda_{\mathrm{SR}}\lambda_{\mathrm{BS}}\rho }{\kappa \Delta }\right)}^{\frac{t+1}{2}}\frac{2m\lambda_{\mathrm{RD}}}{m\left(\lambda_{\mathrm{SR}}+\lambda_{\mathrm{RD}}\right)-\lambda_{\mathrm{SR}}}K_{1-t}\left(2\sqrt{\frac{\lambda_{\mathrm{SR}}\lambda_{\mathrm{BS}}\rho }{\kappa \Delta }}\right)\\ & +\sum_{t=0}^{K-1}\sum_{m=1}^{M}{\left(-1\right)}^{m-1}\frac{C_{M}^{m}}{t!}{\left(\frac{m\left(\lambda_{\mathrm{SR}}+\lambda_{\mathrm{RD}}\right)\lambda_{\mathrm{BS}}\rho }{\kappa \Delta }\right)}^{\frac{t+1}{2}}\\ & \times \frac{2\left(m-1\right)\lambda_{\mathrm{SR}}}{m\left(\lambda_{\mathrm{SR}}+\lambda_{\mathrm{RD}}\right)-\lambda_{\mathrm{SR}}}K_{1-t}\left(2\sqrt{\frac{m\left(\lambda_{\mathrm{SR}}+\lambda_{\mathrm{RD}}\right)\lambda_{\mathrm{BS}}\rho }{\kappa \Delta }}\right)\\ & +\sum_{t=0}^{K-1}\sum_{n=1}^{N}\sum_{m=1}^{M}{\left(-1\right)}^{m+n-1}\frac{C_{N}^{n}C_{M}^{m}}{t!}{\left(\frac{\lambda_{\mathrm{BS}}\rho }{\kappa \Delta }\right)}^{\frac{t+1}{2}}\;{\left(\frac{\rho }{n\lambda_{\mathrm{SP}}\delta \Delta +\lambda_{\mathrm{SR}}\rho }\right)}^{\frac{1-t}{2}}\\ & \times \frac{2m\lambda_{\mathrm{SR}}\lambda_{\mathrm{RD}}}{m\left(\lambda_{\mathrm{SR}}+\lambda_{\mathrm{RD}}\right)-\lambda_{\mathrm{SR}}}K_{1-t}\left(2\sqrt{\frac{\lambda_{\mathrm{BS}}}{\kappa \Delta }\left(n\lambda_{\mathrm{SP}}\delta \Delta +\lambda_{\mathrm{SR}}\rho \right)}\right)\\ & +\sum_{t=0}^{K-1}\sum_{n=1}^{N}\sum_{m=1}^{M}{\left(-1\right)}^{m+n-1}\frac{C_{N}^{n}C_{M}^{m}}{t!}{\left(\frac{\lambda_{\mathrm{BS}}\rho }{\kappa \Delta }\right)}^{\frac{t+1}{2}}\;{\left(\frac{\rho }{n\lambda_{\mathrm{SP}}\delta \Delta +m\left(\lambda_{\mathrm{SR}}+\lambda_{\mathrm{RD}}\right)\rho }\right)}^{\frac{1-t}{2}}\\ & \times \;\frac{2m\left(m-1\right)\lambda_{\mathrm{SR}}\left(\lambda_{\mathrm{SR}}\;+\;\lambda_{\mathrm{RD}}\right)}{m\left(\lambda_{\mathrm{SR}}\;+\;\lambda_{\mathrm{RD}}\right)\;-\;\lambda_{\mathrm{SR}}}K_{1-t}\;\;\left(\;2\sqrt{\frac{\lambda_{\mathrm{BS}}}{\kappa \Delta }\left(n\lambda_{\mathrm{SP}}\delta \Delta +m\left(\lambda_{\mathrm{SR}}+\lambda_{\mathrm{RD}}\right)\rho \right)}\right)\;. \end{array}$$

Similarly, we can obtain a closed-form expression for $\mathrm{Pr}\left(Z_{2}\geq \frac{\rho }{\Delta \gamma_{R_{c}D}}\right),$ and then submit the obtained results into (71) to finish the proof. ☐

### 3.2. Throughput

The throughput (TP) of the H-PRS, C-ORS and B-ORS protocols can be obtained by substituting the expressions of the outage probability (OP) into (19).

## 4. Simulation Results

In this section, a set of numerical results are presented to illustrate the performances of three proposed EH DF cooperative relay selection schemes under the interference constraints of multiple PUs. Monte-Carlo simulations are utilized to verify the theoretical derivations. In the simulation environment, the network nodes are arranged in Cartesian coordinates, where the node S is located at the origin. In addition, the coordinates of the relays, destination, beacon, and primary users are $\left(x_{R},0\right),\;\left(1,0\right),\;\left(0.5,0.5\right),\;\left(x_{P},y_{P}\right),$ respectively. In all of the simulations, we fix the path-loss exponent, the ratio between $I_{\mathrm{th}}$ and $P_{B}$, the energy conversion efficiency, total time of each data transmission, the number of primary users, and the number of antennas at the power beacon by β=3, μ=0.25, η=1, T=1, N=2 and K=2, respectively. Note that, in all simulation results, the simulation results (Sim), the exact theoretical results (Exact) and the asymptotically theoretical results (Asym) are denoted by markers, solid line, and dash line, respectively.

**Figure 2:**
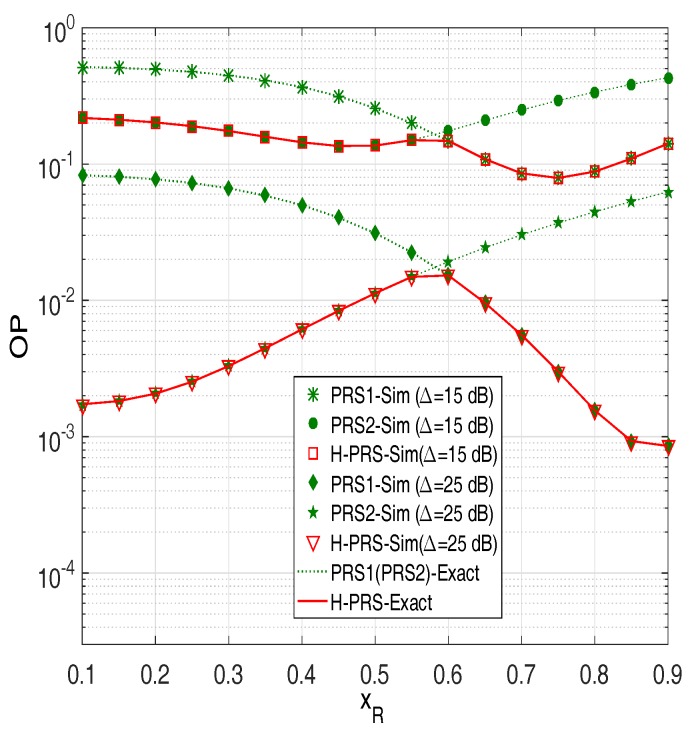
Outage probability of the PRS protocols as a function of $x_{R}$ when M=3, $x_{P}=0.5,$ $y_{P}=-0.5,$ α=0.2, $C_{\mathrm{th}}=0.5,$ $\tau_{D}^{2}=0.1$ and $\tau_{I}^{2}=0.05$.

In Figure 2, we present outage probability (OP) of the conventional PRS protocol [35] (denoted by PRS1), the modified PRS protocol [37] (denoted by PRS2) and the proposed H-PRS protocol as a function of $x_{R}$. This figure shows that the analytical results are in complete agreement with the simulation results. Next, we can see that as $x_{R}$ is small (the relays are close to the source but far from the destination), OP of PRS1 is higher than that of PRS2. However, as $x_{R}$ is high enough, PRS1 outperforms PRS2. As we can see, OP of H-PRS is the same as OP of PRS1, as the relays are near the destination, and is the same as OP of PRS2, as the relays are near the source. Moreover, there exists a value of $x_{R}$ (denoted $x_{R}^{*}$) at which the OP values of PRS1 and PRS2 are same. Indeed, by solving the equation $OP_{\mathrm{PRS}1}=OP_{\mathrm{PRS}2}$ (using (34) and (35)), we can find the value of $x_{R}^{*}$. Finally, it is also seen from Figure 2 that the outage performance of PRS1, PRS2 and H-PRS is better as increasing the transmit SNR (Δ).

**Figure 3:**
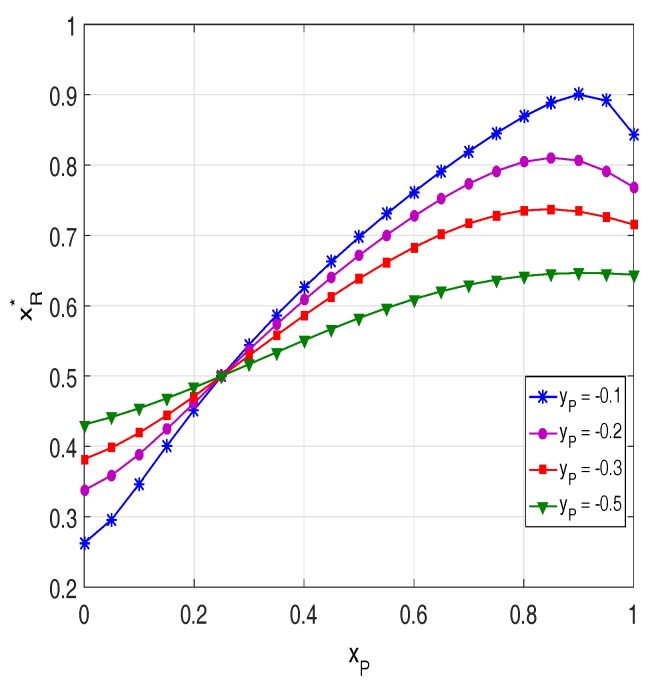
$x_{R}^{*}$ as a function of $x_{P}$ when Δ=15 dB, M=4, α=0.2, $C_{\mathrm{th}}=0.1,$ $\tau_{D}^{2}=0.1$ and $\tau_{I}^{2}=0.05$.

Figure 3 presents the values of $x_{R}^{*}$ with different positions of the primary users. As mentioned above, $x_{R}^{*}$ is obtained by solving the equation $OP_{\mathrm{PRS}1}=OP_{\mathrm{PRS}2}$. Moreover, $x_{R}^{*}$ is a reference distance (between the source and the relays) used in H-PRS to determine which protocol (PRS1 or PRS2) will be used to send the source data to the destination. Particularly, as $x_{R}<x_{R}^{*}$, PRS2 is employed and as $x_{R}>x_{R}^{*}$, the PRS1 is used. As observed from Figure 3, the position of the primary users has a significant impact on $x_{R}^{*}$. It is seen that, when the primary users are close to the source ($x_{P}$ is small), the value of $x_{R}^{*}$ is low and vice versa.

**Figure 4:**
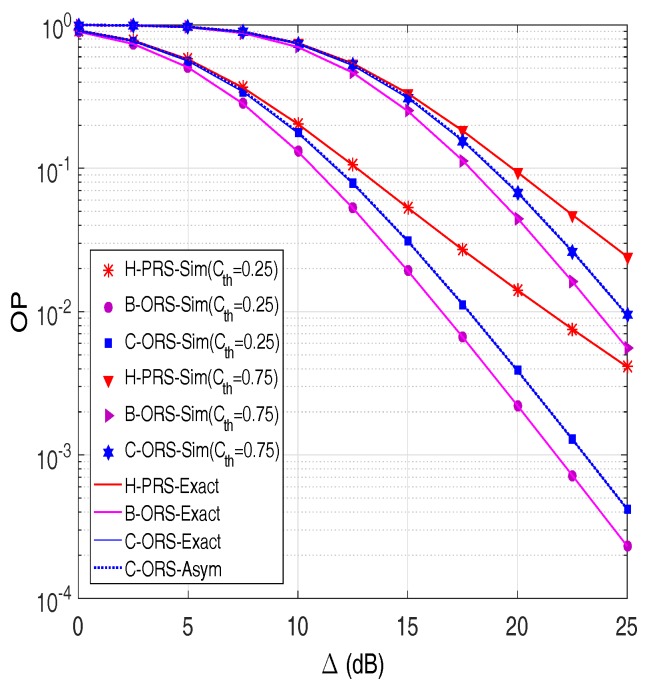
Outage probability as a function of Δ in dB when M=2, $x_{R}=0.5,$ $x_{P}=0.5,$ $y_{P}=-0.5,$ α=0.25, and $\tau_{D}^{2}=\tau_{I}^{2}=0.$

Figure 4 compares the outage performance of H-PRS, B-ORS and C-ORS with various values of $C_{\mathrm{th}}$. We can see that the OP of B-ORS is lowest, and the OP of H-PRS is highest. At high transmit SNR, OP of B-ORS and C-ORS rapidly decrease as Δ is increasing. It is due to the fact that B-ORS and C-ORS obtain a higher diversity gain as compared with H-PRS.

**Figure 5:**
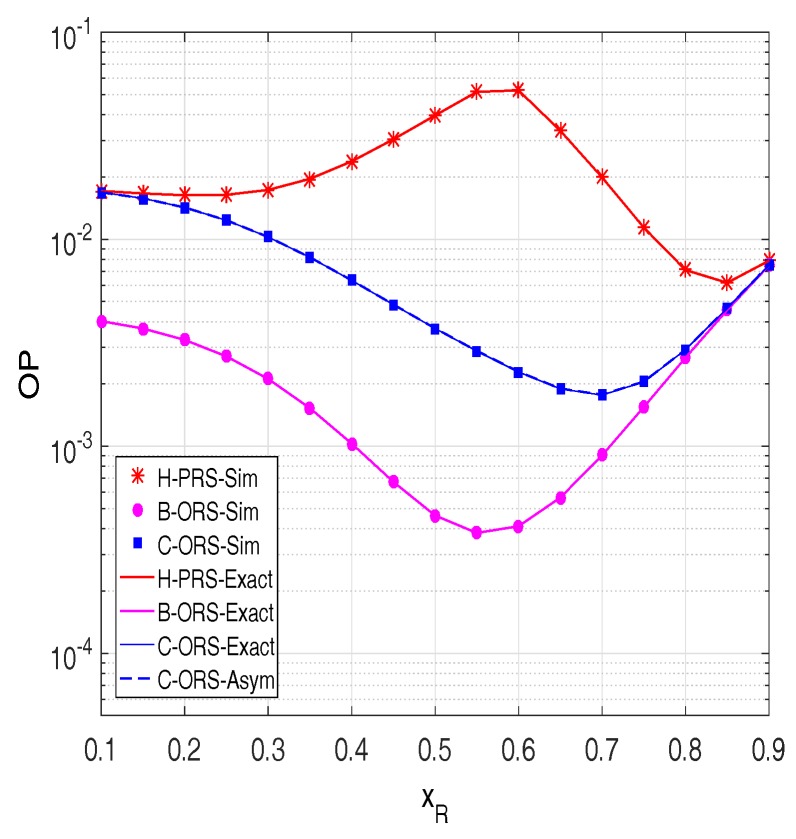
Outage probability as a function of $x_{R}$ when Δ=20 dB, M=4, $x_{P}=0.5,$ $y_{P}=-0.5,$ α=0.1, $C_{\mathrm{th}}=0.6,$ $\tau_{D}^{2}=0.1$ and $\tau_{I}^{2}=0.05.$

In order to investigate the impact of distances on the outage performance of the proposed protocols, we present OP as a function of the locations of the relays on the *x*-axis ($x_{R}$). Figure 5 shows that there exists an optimal position of the relays, at which the OP value of B-ORS and C-ORS is lowest. For H-PRS, its performance is similar to the performance of C-ORS when the relays are near the source. In addition, an interesting result can be observed that when the relays are near the destination, the OP value of H-PRS reaches that of B-ORS and C-ORS. This can be explained by the fact that when the relays are very close to the destination, OP of all of the protocols significantly depends on the source to relay link, thus H-PRS can be roughly approximated to B-ORS and C-ORS. However, different from B-ORS and C-ORS, the performance of H-PRS is not good as the relays are in the middle of the source and the destination, e.g., OP of H-PRS is highest when $x_{R}$ is about 0.6.

**Figure 6:**
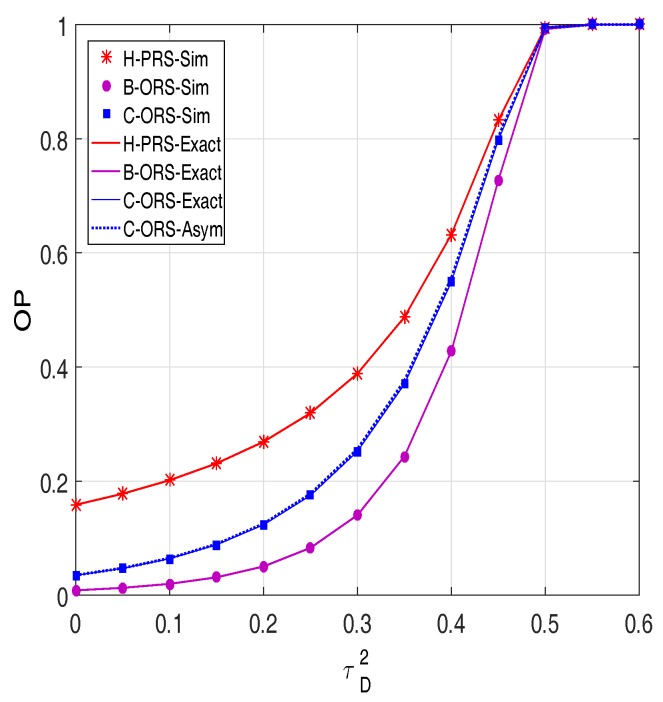
Outage probability as a function of $\tau_{D}^{2}$ when Δ=15 dB, M=5, $x_{R}=0.6,$ $x_{P}=0.5,$ $y_{P}=-0.5,$ α=0.1, $C_{\mathrm{th}}=0.7,$ and $\tau_{I}^{2}=\tau_{D}^{2}/2.$

In Figure 6, we investigate the impact of the hardware impairment level $\left(\tau_{D}^{2}\right)$ on the performance of H-PRS, B-ORS and C-ORS. As we can see, the OP values rapidly increase with the increasing of $\tau_{D}^{2}$. Moreover, Figure 6 shows that all of the proposed protocols are always in outage when $\tau_{D}^{2}$ is higher than 0.55. As stated in Section 3, if $\tau_{D}^{2}\geq 0.55,$ then $1-\tau_{D}^{2}\theta <0,$ and hence $OP_{H-\mathrm{PRS}}=OP_{B-\mathrm{ORS}}=OP_{C-\mathrm{ORS}}=1$.

**Figure 7:**
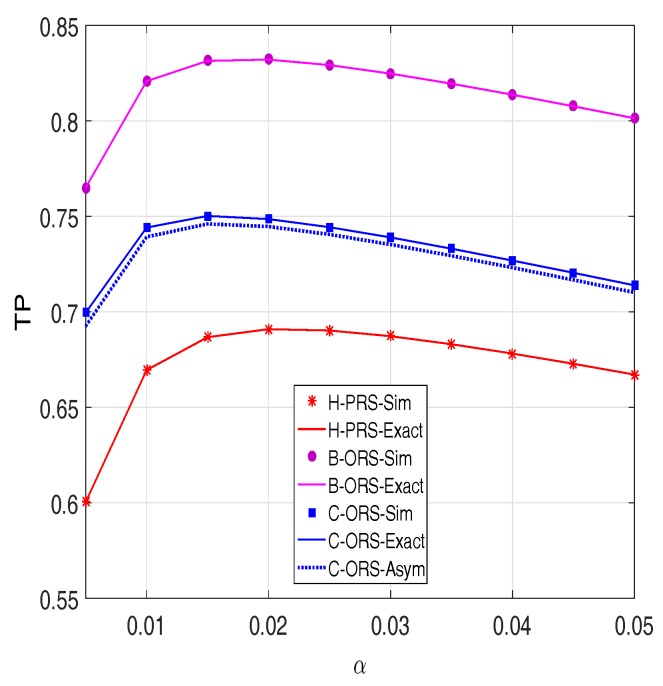
Throughput as a function of α when Δ=15 dB, M=3, $x_{R}=0.5,$ $x_{P}=0.5,$ $y_{P}=-0.5,$ $C_{\mathrm{th}}=1,$ and $\tau_{I}^{2}=\tau_{D}^{2}=0.$

In Figure 7, the throughput (TP) is presented as a function of the fraction of time allocated for the EH process. As presented in the previous sections, the α value plays a key role in the EH process, since it affects both the harvested power, and the transmit power of the source or the selected relay node. As we can see from this figure, there exist optimal values of α at which the throughput of the proposed protocols is highest. This can be explained as follows when the α value is too small: less energy can be harvested from the power beacon. Hence, the small amount of energy that the source or relay node can use for data transmission. At the other extreme, when the α value is too large, a less effective transmission time is utilized to relay the data from source to destination, which leads to the decreasing of the throughput. Therefore, for practical design, the best TP performance can be obtained when α reaches the optimal value. Finally, similar to the OP metric, for all α values, the TP performance of B-ORS is always better than that of C-ORS, which further outperforms H-PRS.

**Figure 8:**
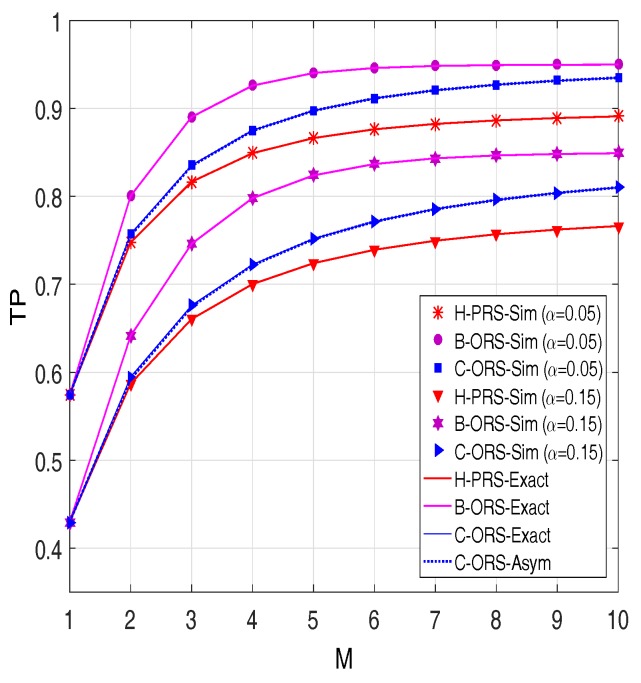
Throughput as a function of *M* when Δ=20 dB, M=3, $x_{R}=0.4,$ $x_{P}=0.5,$ $y_{P}=-0.5,$ $C_{\mathrm{th}}=1,$ $\tau_{D}^{2}=0.1$ and $\tau_{I}^{2}=0.05.$

Figure 8 demonstrates TP versus the number of relays. As expected, the throughput of H-PRS, B-ORS and C-ORS can be enhanced by increasing the *M* value. Again, we can see that the performance of the considered protocols can be improved by assigning the value of α appropriately.

From Figure 4, Figure 5, Figure 6, Figure 7 and Figure 8, it is evident that the simulation results are perfectly consistent with our derived theoretical values, and the gap between the exact and asymptotic results is small, which verifies the correction of our derivations.

## 5. Conclusions

This paper aims to improve the performance of PB-assisted underlay CR in cooperative relaying WSNs under the joint impact of hardware impairments and interference constraint. We have proposed three relaying protocols, where the multi-antenna PB is employed to power the dual-hop DF relaying operation. We derived the exact and asymptotic expressions of the outage probability and throughput of the proposed protocols under the presence of multiple PUs, and over i.i.d. Rayleigh fading channels. The numerical results showed that the performance improvements of B-ORS are higher than those of C-ORS, which, in turn, outperforms H-PRS. Finally, the system performance of the proposed protocols can be enhanced by setting an appropriate energy-harvesting ratio, increasing the number of relays, and placing the relays at the advisable position. 

## References

[1] Lin H., Bai D., Gao D., Liu Y. (2016). Maximum data collection rate routing protocol based on topology control for rechargeable wireless sensor networks. Sensors.

[2] Wang Z., Zeng P., Zhou M., Li D., Wang J. (2017). Cluster-based maximum consensus time synchronization for industrial wireless sensor networks. Sensors.

[3] Jiang L., Tian H., Xing Z., Wang K., Zhang K., Maharjan S., Gjessing S., Zhang Y. (2016). Social-aware energy harvesting device-to-device communications in 5G networks. IEEE Wirel. Commun..

[4] Yadav A., Goonewardena M., Ajib W., Dobre O.A., Elbiaze H. (2017). Energy management for energy harvesting wireless sensors with adaptive retransmission. IEEE Trans. Commun..

[5] Paradiso J.A., Starner T. (2005). Energy scavenging for mobile and wireless electronics. IEEE Pervasive Comput..

[6] Raghunathan V., Ganeriwal S., Srivastava M. (2006). Emerging techniques for long lived wireless sensor networks. IEEE Commun. Mag..

[7] Hieu T.D., Dung L.T., Kim B.S. (2016). Stability-aware geographic routing in energy harvesting wireless sensor networks. Sensors.

[8] Varshney L.R. Transporting Information and Energy Simultaneously. Proceedings of the IEEE International Symposium on Information Theory (ISIT).

[9] Zhang R., Ho C.K. (2013). MIMO broadcasting for simultaneous wireless information and power transfer. IEEE Trans. Wirel. Commun..

[10] Zhou X., Zhang R., Ho C.K. (2013). Wireless information and power transfer: architecture design and rate-energy trade-off. IEEE Trans. Commun..

[11] Nasir A.A., Zhou X., Durrani S., Kennedy R.A. (2013). Relaying protocols for wireless energy harvesting and information processing. IEEE Trans. Wirel. Commun..

[12] Shi Q., Liu L., Xu W., Zhang R. (2014). Joint transmit beamforming and receive power splitting for MISO SWIPT systems. IEEE Trans. Wirel. Commun..

[13] Krikidis I. (2014). Simultaneous information and energy transfer in large scale networks with/without relaying. IEEE Trans. Commun..

[14] Huang K., Lau V.K.N. (2014). Enabling wireless power transfer in cellular networks: Architecture, modeling and deployment. IEEE Trans. Wirel. Commun..

[15] Le N.P. (2018). Throughput analysis of power-beacon assisted energy harvesting wireless systems over non-identical Nakagami-m fading channels. IEEE Commun. Lett..

[16] Liu Y., Wang L., Zaidi S.A.R., Elkashlan M., Duong T.Q. (2016). Secure D2D communication in large-scale cognitive cellular networks: A wireless power transfer model. IEEE Trans. Commun..

[17] Doan T.X., Hoang T.M., Duong T.Q., Ngo H.Q. (2017). Energy harvesting-based D2D communications in the presence of interference and ambient RF sources. IEEE Access.

[18] Tehrani M.N., Uysal M., Yanikomeroglu H. (2014). Device-to-device communication in 5G cellular networks: Challenges, solutions, and future directions. IEEE Commun. Mag..

[19] Van N.T., Duy T.T., Hanh T., Bao V.N.Q. Outage Analysis of Energy-Harvesting Based Multihop Cognitive Relay Networks with Multiple Primary Receivers and Multiple Power Beacons. Proceedings of the International Symposium on Antennas and Propagation (ISAP).

[20] Van N.T., Do T.N., Bao V.N.Q., An B. (2018). Performance analysis of wireless energy harvesting multihop cluster-based networks over Nakgami-m fading channels. IEEE Access.

[21] Hieu T.D., Duy T.T., Choi S.G. Performance Enhancement for Harvest-to-Transmit Cognitive Multi-Hop Networks with Best Path Selection Method Under Presence of Eavesdropper. Proceedings of the IEEE 20th International Conference on Advanced Communication Technology (ICACT).

[22] Hieu T.D., Duy T.T., Kim B.S. (2018). Performance enhancement for multi-hop harvest-to-transmit WSNs with path-selection methods in presence of eavesdroppers and hardware noises. IEEE Sens. J..

[23] Mitola J., Maguire G.Q. (1999). Cognitive radio: Making software radios more personal. IEEE Pers. Commun..

[24] Kong F., Cho J., Lee B. (2017). Optimizing spectrum sensing time with adaptive sensing interval for energy-efficient CRSNs. IEEE Sens. J..

[25] Dung L.T., Hieu T.D., Choi S.G., Kim B.S., An B. (2017). Impact of beamforming on the path connectivity in cognitive radio ad-hoc networks. Sensors.

[26] Joshi G.P., Nam S.Y., Kim S.W. (2013). Cognitive radio wireless sensor networks: Applications, challenges and research trends. Sensors.

[27] Wu Y., Cardei M. (2016). Multi-channel and cognitive radio approaches for wireless sensor networks. Comput. Commun..

[28] Guo Y., Kang G., Zhang N., Zhou W., Zhang P. (2010). Outage performance of relay-assisted cognitive-radio system under spectrum sharing constraints. Electron. Lett..

[29] Lee J., Wang H., Andrews J.G., Hong D. (2011). Outage probability of cognitive relay networks with interference constraints. IEEE Trans. Wirel. Commun..

[30] Laneman J.N., Tse D.N., Wornell G.W. (2004). Cooperative diversity in wireless networks: Efficient protocols and outage behavior. IEEE Trans. Inf. Theory.

[31] Bletsas A., Khisti A., Reed D.P., Lippman A. (2006). A simple cooperative diversity method based on network path selection. IEEE J. Sel. Areas Commun..

[32] Dongyang X., Pinyi R., Qinghe D., Li S. (2015). Joint dynamic clustering and user scheduling for downlink cloud radio access network with limited feedback. China Commun..

[33] Dongyang X., Du Q., Ren P., Sun L., Zhao W., Hu Z. AF-Based CSI Feedback for User Selection in Multi-User MIMO Systems. Proceedings of the IEEE Global Communication Conference (GLOBECOM).

[34] Tourki K., Yang H.C., Alouini M.S. (2011). Accurate outage analysis of incremental decode-and-forward opportunistic relaying. IEEE Trans. Wirel. Commun..

[35] Krikidis I., Thompson J., McLaughlin S., Goertz N. (2008). Amplify-and-forward with partial relay selection. IEEE Commun. Lett..

[36] Ding H., Ge J., da Costa D.B., Jiang Z. (2010). Diversity and coding gains of fixed-gain amplify-and-forward with partial relay selection in Nakagami-m fading. IEEE Commun. Lett..

[37] Duy T.T., Kong H.Y. (2013). Performance analysis of incremental amplify-and-forward relaying protocols with nth best partial relay selection under interference constraint. Wirel. Pers. Commun..

[38] Fredj K.B., Aissa S. (2012). Performance of amplify-and-forward systems with partial relay selection under spectrum-sharing constraints. IEEE Trans. Wirel. Commun..

[39] Sharma P.K., Upadhyay P.K. (2016). Cognitive relaying with transceiver hardware impairments under interference constraints. IEEE Commun. Lett..

[40] Tourki K., Qaraqe K.A., Alouini M.-S. (2013). Outage analysis for underlay cognitive networks using incremental regenerative relaying. IEEE Trans. Veh. Technol..

[41] Hakim H., Boujemaa H., Ajib W. (2013). Performance comparison between adaptive and fixed transmit power in underlay cognitive radio networks. IEEE Trans. Commun..

[42] Hoang D.T., Niyato D., Wang P., Kim D.I. (2014). Opportunistic channel access and RF energy harvesting in cognitive radio networks. IEEE J. Sel. Areas Commun..

[43] Hoang D.T., Niyato D., Wang P., Kim D.I. (2015). Performance analysis of wireless energy harvesting cognitive radio networks under smart jamming attacks. IEEE Trans. Cogn. Commun. Netw..

[44] Nguyen D.K., Jayakody D.N.K., Chatzinotas S., Thompson J.S., Li J. (2017). Wireless energy harvesting assisted two-way cognitive relay networks: Protocol design and performance analysis. IEEE Access.

[45] Xu C., Zheng M., Liang W., Yu H., Liang Y.C. (2016). Outage performance of underlay multihop cognitive relay networks with energy harvesting. IEEE Commun. Lett..

[46] Xu C., Zheng M., Liang W., Yu H., Liang Y.C. (2017). End-to-end throughput maximization for underlay multi-hop cognitive radio networks with RF energy harvesting. IEEE Trans. Wirel. Commun..

[47] Mokhtar M., Gomaa A., Al-Dhahir N. (2013). OFDM AF relaying under I/Q imbalance: Performance analysis and baseband compensation. IEEE Trans. Commun..

[48] Bjornson E., Matthaiou M., Debbah M. (2013). A new look at dual-hop relaying: Performance limits with hardware impairments. IEEE Trans. Commun..

[49] Duy T.T., Duong T.Q., da Costa D.B., Bao V.N.Q., Elkashlan M. (2015). Proactive relay selection with joint impact of hardware impairment and co-channel interference. IEEE Trans. Commun..

[50] Peng C., Li F., Liu H. (2017). Wireless energy harvesting two-way relay networks with hardware impairments. Sensors.

[51] Zhang C., Ge J., Li J., Gong F., Ji Y., Farah M.A. (2016). Energy efficiency and spectral efficiency trade-off for asymmetric two-way AF relaying with statistical CSI. IEEE Trans. Veh. Technol..

[52] Choi J. (2017). Joint rate and power allocation for NOMA with statistical CSI. IEEE Trans. Commun..

[53] Gradshten I.S., Ryzhik I.M. (2007). Table of Integrals, Series and Products.

